# Novel Thiazolo[5,4-*b*]phenothiazine Derivatives: Synthesis, Structural Characterization, and In Vitro Evaluation of Antiproliferative Activity against Human Leukaemia

**DOI:** 10.3390/ijms18071365

**Published:** 2017-06-26

**Authors:** Balazs Brem, Emese Gal, Luiza Găină, Luminiţa Silaghi-Dumitrescu, Eva Fischer-Fodor, Ciprian Ionuţ Tomuleasa, Adriana Grozav, Valentin Zaharia, Lorena Filip, Castelia Cristea

**Affiliations:** 1 Faculty of Chemistry and Chemical Engineering, Babeş-Bolyai University, 400028 Cluj-Napoca, Romania; bbrem@chem.ubbcluj.ro (B.B.); emese@chem.ubbcluj.ro (E.G.); gluiza@chem.ubbcluj.ro (L.G.); lusi@chem.ubbcluj.ro (L.S.-D.); 2 Tumor Biology Department, Ion Chiricuta Oncology Institute, 400015 Cluj-Napoca, Romania; efischerfodor@yahoo.com (E.F.-F.); ciprian.tomuleasa@umfcluj.ro (C.I.T.); 3 Medfuture Research Center, Iuliu Hatieganu University of Medicine and Pharmacy, 400012 Cluj-Napoca, Romania; 4 Research Center for Functional Genomics and Translational Medicine, Iuliu Hatieganu University of Medicine and Pharmacy, 400012 Cluj-Napoca, Romania; 5 Faculty of Pharmacy, Iuliu Hatieganu University of Medicine and Pharmacy, 400012 Cluj-Napoca, Romania; adriana.ignat@umfcluj.ro (A.G.); vzaharia@umfcluj.ro (V.Z.)

**Keywords:** phenothiazine, thiazole, antiproliferative activity, structure-activity relationship

## Abstract

The molecular frame of the reported series of new polyheterocyclic compounds was intended to combine the potent phenothiazine and benzothiazole pharmacophoric units. The synthetic strategy applied was based on oxidative cyclization of *N*-(phenothiazin-3-yl)-thioamides and it was validated by the preparation of new 2-alkyl- and 2-aryl-thiazolo[5,4-*b*]phenothiazine derivatives. Optical properties of the series were experimentally emphasized by UV-Vis absorption/emission spectroscopy and structural features were theoretically modelled using density functional theory (DFT). In vitro activity as antileukemic agents of thiazolo[5,4-*b*]phenothiazine and *N*-(phenothiazine-3-yl)-thioamides were comparatively evaluated using cultivated HL-60 human promyelocytic and THP-1 human monocytic leukaemia cell lines. Some representatives proved selectivity against tumour cell lines, cytotoxicity, apoptosis induction, and cellular metabolism impairment capacity. 2-Naphthyl-thiazolo[5,4-*b*]phenothiazine was identified as the most effective of the series by displaying against THP-1 cell lines a cytotoxicity close to cytarabine antineoplastic agent.

## 1. Introduction

Heterocyclic compounds containing nitrogen and sulphur were frequently evidenced in many biological active compounds of both natural and synthetic origins. Two heteroaromatic compounds commonly recognized for a broad spectrum of pharmacological activities are tricyclic phenothiazine (PTZ) [1] and bicyclic benzothiazole (BTA) [2], each of them with individual synthetic representatives sharing high therapeutic potency as antimicrobial, antimalarial, anthelmintic, analgesic, or anti-inflammatory drugs. The antimicrobial activity of the phenothiazinium salts was exploited in medical and environmental research [3], while the electronic properties of the neutral PTZ-based chromophores finely tuned by variable substitution on the peripheral benzene rings [4,5,6] make this a versatile substrate that is recommended for materials scientific investigations.

Recently, the potential anticancer activity of both PTZ and BTA seemed to be of high research interest. PTZ derivatives, commonly highly related to their neuroleptic action, also exhibited anti-proliferative effect, anti-calmodulin (CAM) action, and inhibition of protein kinases or *P*-glycoprotein (Pgp) transport function [7]. The anti-proliferative activity appeared more strongly correlated to the substituents attached to the carbon atoms of the ring, which increased lipophilicity, and less correlated to the nature of the side chain connected to the heterocyclic nitrogen atom [8]. A direct interaction between PTZ derivatives capable of creating hydrogen bonds and Pgp was also documented as crucial for inhibition of the transport function of the protein and increase of cellular chemosensitivity [9]. Synthetic azaphenothiazines and benzo[*a*]phenothiazines presented in vitro anticancer effects on various cell lines [10,11]. Some antipsychotic PTZ drugs showed effects on leukaemia cells [12,13]. BTA derivatives were also evidenced as anticancer agents based on their topoisomerase, microtubule polymerization, or Cytochrome P450 enzyme inhibition activity [14]. Merging the structure of BTA with other heterocycles appeared as a versatile approach in the design of drug-like molecules with a new pharmacological profile, action, or toxicity and therefore, 2-substituted BTA derivatives were encompassed in the design of several novel drug candidates [15,16,17].

As a synthetic target, 2-substituted BTA may be available by alternative routes involving the condensation of 2-aminothiophenols with carbonyl/carboxylic acid derivatives (acyl chlorides, esters, nitriles), or the cyclization of thiobenzanilides, with a wide range of methodologies developed in order to improve the reaction selectivity, purity, and yield [18]. The intramolecular cyclization of anilides appears as a convenient alternative prompted by copper- [19,20,21,22,23,24] or iron-based [25] catalysts.

**Figure 1:**
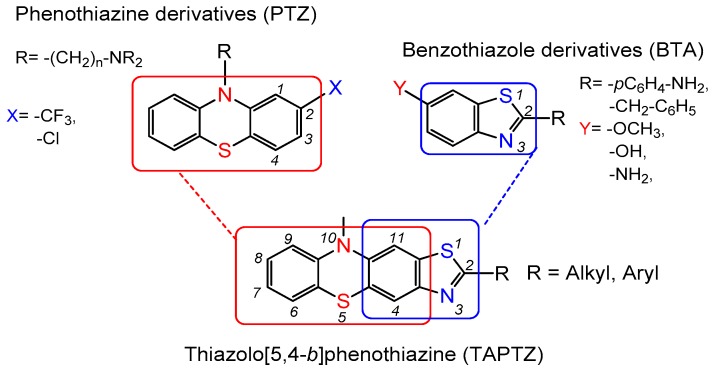
Generic chemical structure and significant substitution pattern for antiproliferative agents derived from PTZ and BTA and their structural complementarities with the target polyheterocyclic TAPTZ structure.

The association of the potent PTZ and thiazole pharmacophores under the same molecular frame was the target of our on-going concern for a rational design of new biologically active compounds. In some of our previous reports, these heterocyclic units appeared separated by a spacer (antitumoral phenothiazinyl-thiazolyl-hydrazine derivatives [26]) or directly joined (antimicrobial benzothiazolyl-phenothiazine derivatives [27,28]). In this work, we described the synthesis and characterization of new representatives of polyheterocyclic compounds containing benzo-fused BTA/PTZ. In the structure of the new 2-substituted-Thiazolo[5,4-*b*]phenothiazine derivatives (TAPTZ), heterocyclic PTZ and BTA units share a central benzene ring and thus offer a similar substitution pattern that is significant for their biological activity. In support of our reasoning, Figure 1 shows the chemical structures of selected PTZ and BTA derivatives with anti-proliferative activity, together with their beneficial overlap in the new TAPTZ derivatives. The in vitro biological experiments described herein were designated to comparatively evaluate the anti-proliferative efficacy of target TAPTZ versus their PTZ precursors as antileukemic agents in human leukaemia cell growth.

## 2. Results

### 2.1. Synthesis of Thiazolo[5,4-b]phenothiazine Derivatives

**Scheme 1:**
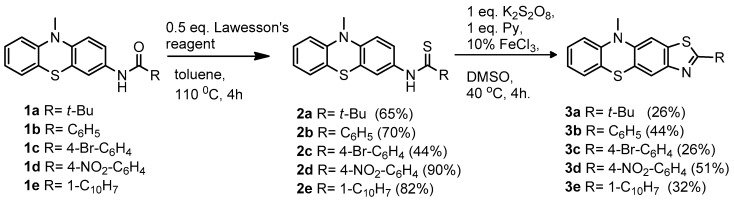
Synthetic path to Thiazolo[5,4-*b*]phenothiazine derivatives TAPTZ.

The synthetic strategy applied for the preparation of the new TAPTZ heterocyclic structure was founded on the selection of PTZ as a building block laid open for assembling the BTA unit, as illustrated in Scheme 1.

The starting *N*-(phenothiazin-3-yl)-amides **1a**–**e**, were prepared from 3-amino-PTZ precursor (conveniently accessible according to our reported microwaves assisted amination procedure [29]) by acylation with different acyl chlorides. The *N*-(phenothiazine-3-yl)-thioamides **2a**–**e** were synthesized in variable yields using Lawesson’s reagent for the conversion of amide functional group into its thio-analogue. In the last step TAPTZ **3a**–**e** were obtained by thiazole ring closure under oxidative conditions [25]. The best cyclization yields were obtained in the synthesis of **3d** by taking benefit of the electron withdrawing effect of the nitro substituent which disabled the competitive conversion to **1d**.

The structure of each new TAPTZ **3a**–**e**, *N*-(phenothiazine-3-yl)-thioamide **2a**–**e** intermediate, and starting amide **1a**–**e** were unambiguously assigned by ^1^H-/^13^C-NMR, UV-vis, and IR spectroscopy and confirmed by high resolution mass spectrometry.(^1^H-/^13^C-NMR and ESI-HRMS spectra of **3a**−**e** are presented in supplementary material). In ^1^H-NMR spectra of **3a**–**e**, the key signals arising from the protons attached to the central benzene ring gave two distinct singlets situated in the regions 7.74–7.84 and 7.18–7.20 ppm, respectively. The more shielded singlet corresponded to the proton situated in the spatial proximity of the *N*-methyl group, as confirmed by homonuclear two-dimensional Nuclear Overhauser Effect Spectroscopy 2D NOESY experiments involving saturation of the transitions corresponding to the three equivalent protons of the methyl group (2D-NOESY spectrum of **3a** is presented in supplementary material). The chemical shift value of the low field singlet appeared slightly influenced by the nature of the substituent attached to the marginal thiazole ring. (NMR spectra of **3a**–**e** are presented in supplementary material).

### 2.2. Spectral Properties

**Figure 2:**
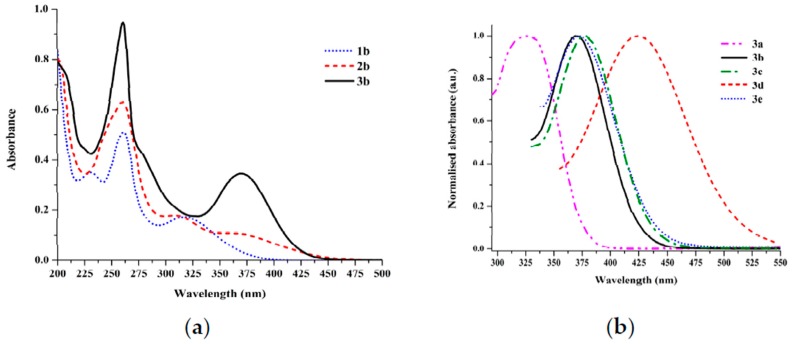
UV Ultraviolet absorption spectra recorded in acetonitrile solution at concentration 10^−5^ M: (**a**) phenyl-TAPTZ **3b**, *N*-(phenothiazine-3-yl)-thiobenzamide **2b**, and *N*-(phenothiazine-3-yl)-benzamide **1b**; (**b**) normalized longest wavelength absorption band for TAPTZ **3a**–**e** (a.u. = arbitrary units).

Each TAPTZ **3a**–**e** exhibited two UV absorption bands situated in the range 250–264 and 326–424 nm, respectively (Table 1). The allowed electronic transitions typical to the phenothiazine core were responsible for the higher energy absorption band also observable in the spectra of *N*-(phenothiazine-3-yl)-thioamides **2a**–**e** and amides **1a**–**e**, respectively, as exemplified in Figure 2a by the overlaid UV absorption spectra of the phenyl substituted derivatives. The second absorption band situated at longer wavelengths was characterized by lower intensity and a strong dependence of its location on the electronic properties of the substituents attached in position 2 of the thiazole unit (Figure 2b). The absorption of *t*-butyl-TAPTZ **3a** occurred at higher energy, while the aromatic substituents enabled a bathochromic shift of 44–98 nm.

**Figure 3:**
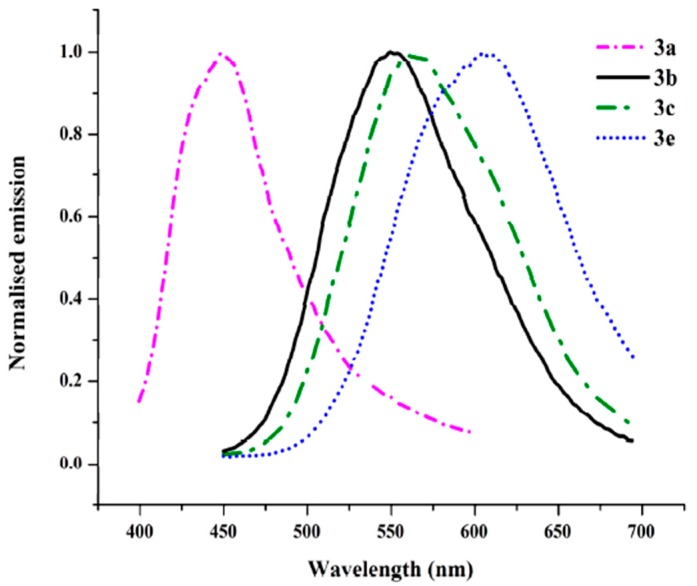
Normalized emission bands of 2-substituted-Thiazolo[5,4-*b*]phenothiazine derivatives **3a**−**3e** (excitation with the corresponding longest wavelength absorption maxima).

In acetonitrile solution, upon excitation with the corresponding longest wavelength absorption maxima, TAPTZ derivatives generated broad, unstructured emission bands situated in the visible range (Figure 3), excepting the most polar nitro-phenyl-TAPTZ **3d**, with emission quenched by dipolar interactions with the solvent. Large Stokes shift values (Table 1) typical to phenothiazine derivatives were detected, suggesting a significant structural reorganization of the polarized excited state as compared to the ground state [30].

### 2.3. Computational Data

**Figure 4:**
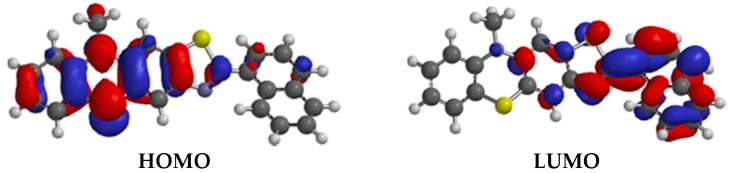
Frontier molecular orbital density plots for TAPTZ **3e** resulted from DFT calculations performed at B3LYP/6-31G* level of theory (the iso-contour value was set to 0.02). The orbital wave functions are positive in regions coloured in red and negative in regions coloured in blue. The ball and stick molecular models display carbon atoms in grey, hydrogen atoms in white, sulfur atoms in yellow and nitorogen atoms in blue.

The molecular structures of the new TAPTZ **3a**–**e** have been studied by using density functional theory (DFT) as implemented in the Spartan 06 software package (Version 06, Wavefunction, Inc., Irvine, CA, USA). The energies of frontier molecular orbitals (FMOs) (E_HOMO_, E_LUMO_) corresponding to the optimized geometry of the lowest energy structure were computed at the B3LYP/6-31G* level of theory and the results were summarized in Table 1. The FMO plots for each TAPTZ indicated that highest occupied molecular orbital HOMO was located on the PTZ core, while lowest unoccupied molecular orbital LUMO included the BTA and the aromatic substituent. Figure 4 shows the frontier molecular orbital density plots for naphthyl-TAPTZ **3e** (frontier molecular orbital density plots for compounds **3a-d** are presented in supplementary material).

**Figure 5:**
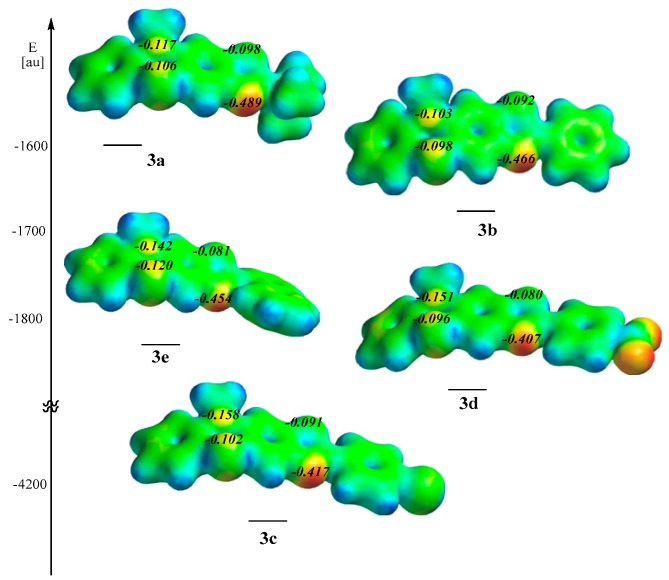
Molecular electrostatic potential surface (EPS) plots of TAPTZ **3a**–**e** hierarchized in order of computed energy of the most stable conformer; **blue** colour on the surface represents highly positive potential regions, **red** colour represents highly negative potentials, while orange, yellow and green colours depict intermediate values of potential. Electrostatic charge is depicted on heteroatoms.

The variation of electron density in TAPTZ **3a**–**e** was modelled by generating the molecular electrostatic potential surface (EPS), as illustrated in Figure 5, which displays the overall molecular shape with electron rich regions coloured in red and electron poor regions in blue colour on the surface. In each case, the heteroatoms of the phenothiazine core shared comparable electron densities, while the thiazole ring appeared much more polarized, accumulating the negative charge on the nitrogen atom, and, consequently, it may be designated as the most probable site for intermolecular electrostatic interactions. The substituent attached in position 2 slightly modulates the electrostatic charge on heteroatoms, as depicted in Figure 5.

The overall polarization of the polyheterocyclic structure expressed in terms of dipole moment is presented in Table 1.

### 2.4. Biological Properties

The antiproliferative activity of the obtained series of *N*-(phenothiazine-3-yl)-thioamides **2a**–**e** and polyheterocyclic TAPTZ **3a**–**e** was investigated in vitro using cultured HL-60 human promyelocytic and THP-1 human monocytic leukaemia cell lines, versus cytarabine, an antineoplastic agent used in the treatment of leukaemia. The cytotoxicity of the studied compounds was assessed using the MTT colorimetric assay and quantified using the parameter median inhibitory concentration (IC_50_); their inhibitory activity against tumour and normal cells in vitro covered a wide range of values, as summarized in Table 2.

Nitro-derivatives **2d**, **3d** did not demonstrate significant growth inhibition against either tumour cells or normal cells. A close inspection of the IC_50_ values listed in Table 2 shows that naphthyl substituted TAPTZ **3e** displayed the most noticeable cytotoxic effect, with an estimated IC_50_ value closest to cytarabine standard against THP-1 and a less pronounced anti-proliferative activity against HL-60 cells, while the corresponding *N*-(phenothiazin-3-yl)-thioamide **2e** seemed completely inactive. None of the compounds with anti-proliferative activity against tumour cells (**3e**, **2a**, **2c**) influenced the survival of the normal peripheral blood mononuclear cells (PBMCs) during the same time period and concentration range, thus proving a marked selectivity.

**Figure 6:**
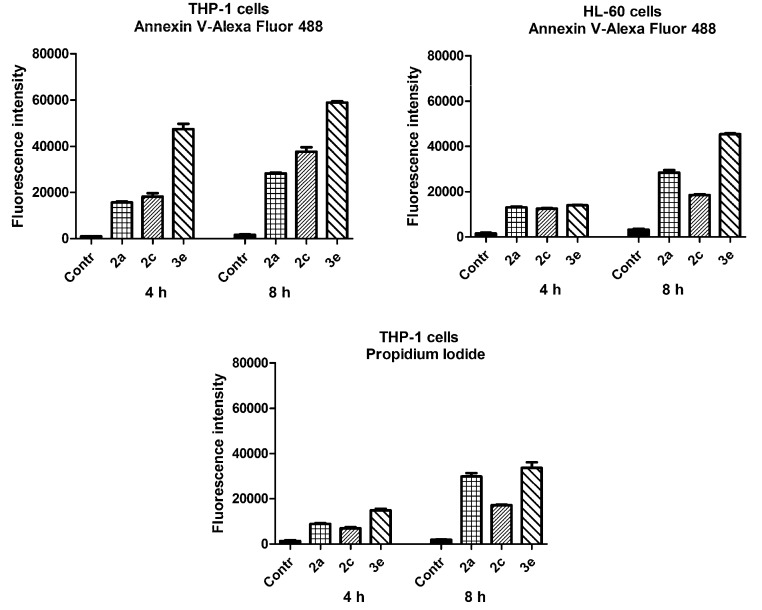
Apoptosis induction capacity of *N*-(phenothiazin-3-yl)-thioamides **2a**, **2c**, and TAPTZ **3e** monitored by fluorescence emission measured at 530–575 nm (488 nm excitation) upon human monocytic leukaemia cell THP-1 and human promyelocytic leukaemia cell HL-60 populations stained with: Annexin V (**above**), propidium iodide (**below**).

The most efficient compounds (**3e**, **2a**, **2c**) were tested for their capacity to trigger the programmed cell death in the leukaemia cell lines (Figure 6). The early apoptotic process was revealed within 4 h after the exposure of the cells to the tested compounds, and the tendency rose when cells were treated for 8 h. A remarkable apoptotic effect was observed for TAPTZ **3e** on both THP-1 and HL-60 cell lines (one-way analysis of variance, Dunnet multiple comparison test, *p* < 0.0001). *N*-(Phenothiazin-3-yl)-pivalamide **2a** was also effective against both cell lines. The parallel measurement of necrotic cells stained with propidium iodide (PI) also displayed the largest number of necrotic cells in populations treated with TAPTZ **3e**, while **2a** caused necrosis mainly in THP-1 cells. The apoptosis induction capacity of **2c**, although inferior to **3e** in THP-1 cells (one-way analysis of variance ANOVA, Bonferroni post-test, *p* < 0.001) and at the 8-h time point even in HL-60 cells, is well balanced by the low proportion of necrotic cells, showing a prevalence to programmed cell death induction. A similar behaviour of PTZ derivatives was previously mentioned with respect to compound selectivity towards normal lymphocytes [31,32] and therefore we presume that the mechanism of apoptosis induction in treated THP-1 and HL-60 cells may be associated with the inhibition of mitochondrial DNA polymerase, decreased ATP production, and caspases fragmentation.

The alteration of cell reducing capacity after the treatment with *N*-(phenothiazin-3-yl)-thioamides in series **2** and TAPTZ in series **3** was evaluated based on Alamar Blue stain reduction in viable cells, as an indicator of cellular metabolism impairment [33] (supplementary material Figure S1). The THP-1 cell reducing capacity diminished significantly after treatment with **2a**, **2c**, **3a**, **3c**, **3e**. Compounds **2c**, **3a**, **3b**, **3e** showed similar activity against HL-60 populations, and according to the correlation with the minor modifications of PBMC viability, none of the tested compounds had the capacity to influence the metabolism of the normal leukocytes. It is known that PTZ influence the drug transportation through Pgp pumps [34] and therefore the metabolic alteration can be related to this capacity. Compounds with significant cytotoxicity **2c**, **3e** against tested cell lines typically affected the cellular metabolic activity as well, while **2a** (which presented a lower toxicity upon HL-60 populations) did not influence the reducing environment of the cells, even though its capability to induce apoptosis was evidenced.

## 3. Discussion

The described synthetic protocol offers a reliable route to the heterocyclic system containing benzo-fused thiazole and phenothiazine units with linear arrangement. Apart from the preparation of various 2-substituted TAPTZ derivatives, the scope of this methodology can be broadened to the preparation of novel constitutional isomers of TAPTZ when starting with different position isomers of the amino-phenothiazine precursors.

The optical absorption and emission properties of TAPTZ derivatives appear slightly modulated by the nature of the substituent attached to the thiazole unit. UV-Vis absorption and emission spectroscopy can be conveniently employed in the detection of the TAPTZ derivatives in various environments. The large Stokes shift values recommend TAPTZ derivatives approaching near IR emission maxima (e.g., **3e**) for imagistic applications.

The tested series of TAPTZ derivatives and *N*-(phenothiazine-3-yl)-thioamides displayed generally modest biological activity. Based on structural similarity with previously reported PTZ derivatives with anti-proliferative activity, TAPTZs emphasized a favourable substitution pattern of the PTZ core induced by the fusion of the thiazole ring, possible hydrogen bond associations involving mainly the nitrogen atom of the thiazole unit, and biological activity increased by the presence of hydrophobic substituents.

## 4. Materials and Methods

All chemicals used were of reagent grade. The reaction progress was monitored by thin layer chromatography on Merck DC Alufolien, silica gel 60 F_254_ and components were visualized by UV lamp VL-4LC. Purification by flash chromatography was performed on silica gel 60 (particle size 0.032–0.063 mm) and recrystallization.

EI-MS spectra were recorded on a GC-MS QP 2010 Shimadzu mass spectrometer and HRMS spectra on Thermo LTQ Orbitrap XL. NMR spectra were recorded at room temperature in solution on 400 or 600 MHz Bruker Avance instruments. Chemical shifts are expressed in terms of δ (ppm) relative to standard tetramethylsilane (TMS).

### 4.1. General Procedure for Obtaining N-Acyl-3-Aminophenothiazines *(**1a**–**e**)*

To a mixture of 3-amino-10-methyl-10*H*-phenothiazine [22] (6.5 mmol) and TEA (7 mmol) in CH_2_Cl_2_ (20 mL) at 0 °C and under inert atmosphere was added the corresponding acyl-chloride (6.5 mmol). After stirring at room temperature for 12 h, the reaction mixture was concentrated and further diluted with brine (50 mL). The two phases were separated and the water phase was extracted with dichloromethane (3 × 100 mL). The combined organic phases were dried and the solvent was evaporated. The crude product was subjected to column chromatography (silica gel, toluene, or toluene/acetone = 10/1) which provided **1a**–**e** as white or cream powder.

*N-(10-Methyl-10H phenothiazin-3-yl)pivalamide* (**1a**). Purification by flash chromatography gave **1a** (1.6 g, 80%) as white solid, m.p. 188–190 °C. ^1^H-NMR (400 MHz; DMSO-*d*_6_; Me_4_Si): δ_H_ 1.19 (9H, s, CH_3_), 3.26 (3H, s, NCH_3_), 6.87 (1H, d, ^3^*J* = 8.7 Hz, 1-H), 6.91–6.95 (2H, m, 9-H, 7-H), 7.14 (1H, d, ^3^*J* = 7.3 Hz, 6-H), 7.19 (1H, t, ^3^*J* = 8.0 Hz, 8-H), 7.45 (1H, dd, ^3^*J* = 8.7 Hz, ^4^*J* = 2.0 Hz, 2-H), 7.5 (1H, d, ^4^*J* = 2.0 Hz, 4-H), 9.12 (1H, brs, NH). ^13^C-NMR (100 MHz; DMSO-*d*_6_): δ_C_ 27.2 (3C), 35.0, 39.5, 114.2, 114.3, 118.7, 119.5, 121.7, 121.8, 122.2, 126.7, 127.7, 134.4, 140.7, 145.4, 176.2. HRMS (ESI) *m*/*z*: Calcd. for C_18_H_21_N_2_OS [M + H]^+^ 313.1369; Found 313.1358.

*N-(10-Methyl-10H-phenothiazin-3-yl)benzamide* (**1b**). Purification by flash chromatography gave **1b** (3.78 g, 81%) as grey solid, m.p. 220–222 °C (lit [35]). ^1^H-NMR (400 MHz, DMSO-*d*_6_, Me_4_Si): δ_H_ 3.29 (3H, s, NCH_3_), 6.93–6.96 (3H, m, 9-H, 1-H, 7-H), 7.17 (1H, dd, ^3^*J* = 8.0 Hz, ^4^*J* = 1.4 Hz, 6-H), 7.21 (1H, td, ^3^*J* = 8.5 Hz, ^4^*J* = 1.2 Hz, 8-H), 7.50–7.58 (3H, m, Ph), 7.6 (1H, dd, ^3^*J* = 8.8 Hz, ^4^*J* = 2.2 Hz, 2-H), 7.67 (1H, d, ^4^*J* = 2.2 Hz, H), 7.94 (2H, d, ^3^*J* = 7.0 Hz, Ph,), 10.19 (1H, brs, NH). ^13^C-NMR (100 MHz; DMSO-*d*_6_): δ_C_ 35.5, 114.8 (2C), 119.1, 120.1, 122.1, 122.5, 122.7, 127.2, 128.0 (2C), 128.2, 128.8 (2C), 131.9, 134.7, 135.2, 141.6, 145.8, 165.6. HRMS (ESI) *m*/*z*: Calcd. for C_20_H_17_N_2_OS [M + H]^+^ 333.1055; Found 333.1065.

*N-(10-Methyl-10H-phenothiazin-3-yl)-4-bromobenzamide* (**1c**). Purification by flash chromatography gave **1c** (0.9 g, 56%) as grey solid, m.p. 202–204°C. ^1^H-NMR (400 MHz, DMSO-*d*_6_, Me_4_Si): δ_H_ 3.29 (3H, s, NCH_3_), 6.93–6.95 (3H, m, 9-H, 1-H, 7-H), 7.16 (1H, d, ^3^*J* = 7.0 Hz, 6-H), 7.21 (1H, t, ^3^*J* = 7.3 Hz, 8-H), 7.45 (2H, d, ^3^*J* = 8.1 Hz, Ph), 7.60 (1H, d, ^3^*J* = 8.0 Hz, 2-H), 7.67 (1H, s, 4-H), 7.81 (2H, d, ^3^*J* = 8.12 Hz, Ph) 10.46 (1H, brs, NH). ^13^C-NMR (100 MHz; DMSO-*d*_6_): δ_C_ 35.5, 113.6, 114.4, 118.8, 119.9, 121.6, 122.0, 122.3, 124.2, 126.8, 127.8 (2C), 129.7 (2C), 133.8, 140.8, 141.3, 144.8, 145.3, 164.2. HRMS (ESI) *m*/*z*: Calcd. for C_20_H_16_BrN_2_OS [M + H]^+^ 411.0161/ 413.0140; Found 411.0168/ 413.0145.

*N-(10-Methyl-10H-phenothiazin-3-yl)-4-nitrobenzamide* (**1d**). Purification by flash chromatography gave **1d** (2.21 g, 89%) as grey solid, m.p. 245–247 °C. ^1^H-NMR (400 MHz, DMSO-*d*_6_, Me_4_Si): δ_H_ 3.30 (3H, s, NCH_3_), 6.93–6.97 (3H, m, 1-H, 9-H, 7-H), 7.16 (1H, d, ^3^*J* = 7.0 Hz, 6-H), 7.21 (1H, t, ^3^*J* = 7.3 Hz, 8-H), 7.60 (1H, d, ^3^*J* = 8.0 Hz, 2-H), 7.67 (1H, s, 4-H), 8.16 (2H, d, ^3^*J* = 8.1 Hz, Ph), 8.35 (2H, d, ^3^*J* = 8.1 Hz, Ph) 10.50 (1H, brs, NH). ^13^C-NMR (100 MHz; DMSO-*d*_6_): δ_C_ 35.5, 114.4 (2C), 118.8, 119.8, 121.8, 122.1, 122.3, 123.5 (2C), 126.8, 127.8, 129.1 (2C), 133.7, 140.4, 141.6, 145.2, 149.1, 163.4. HRMS (ESI) *m*/*z*: Calcd. for C_20_H_16_N_3_O_3_S [M + H]^+^ 378.0907; Found 378.0899.

*N-(10-Methyl-10H-phenothiazin-3-yl)-1-naphthamide* (**1e**). Purification by flash chromatography gave **1e** (1.37 g, 60%) as grey solid, m.p. 230–232 °C. ^1^H-NMR (400 MHz, DMSO-*d*_6_, Me_4_Si): δ_H_ 3.31 (3H, s, NCH_3_), 6.94–6.98 (3H, m, 9-H, 1-H, 7-H), 7.18 (1H, d, ^3^*J* = 7.0 Hz, 6-H), 7.22 (1H, t, ^3^*J* = 7.5 Hz, 8-H), 7.58–7.63 (3H, m, 4-H, Napth), 7.64 (1H, dd, ^3^*J* = 8.7 Hz, ^4^*J* = 2 Hz, 2-H), 7.74–7.75 (2H, m, Naph), 8.01 (1H, m, Naph), 8.05 (1H, d, ^3^*J* = 8.2 Hz, Naph), 8.21 (1H, m, Naph), 10.53 (1H, brs, NH). ^13^C-NMR (100 MHz; DMSO-*d*_6_): δ_C_ 35.5, 114.4, 114.5, 118.2, 119.1, 121.6, 122.2, 122.3, 125.0, 125.1, 125.4, 126.3, 127.0, 127.8, 128.3, 129.6, 130.1, 130.6, 133.1, 134.4, 134.6, 141.2, 145.4, 166.9. HRMS (ESI) *m*/*z*: Calcd. for C_24_H_19_N_2_OS [M + H]^+^ 383.1213; Found 383.1232.

### 4.2. General Procedure for Obtaining N-(Phenothiazin-3-yl)tioamides *(**2a**–**e**)*

The amide **1a**–**e** (1mmol) and the Lawesson’s reagent [2,4-bis(4-methoxyphenyl)-1,3,2,4-dithiadiphosphetane]-2,4-dithione (0.5 mmol)] were heated to reflux in toluene (70 mL). A clear solution was quickly formed; after a 4 h reflux period the reaction mixture was concentrated. The crude product was purified by flash chromatography (silica gel, toluene, or toluene/ethyl acetate = 20/1) to afford compounds **2a**–**e** as yellowish powders.

*N-(10-Methyl-10H-phenothiazin-3-yl)pivalthioamide* (**2a**). Purification by flash chromatography gave **2a** (0.7 g, 65%) as yellow solid, m.p. 138–140 °C. ^1^H-NMR (400 MHz, DMSO-*d*_6_, Me_4_Si): δ_H_ 1.44 (9H, s, *t*-Bu), 3.34 (3H, s, NCH_3_), 6.76 (1H, d, ^3^*J* = 8.6 Hz, 1-H), 6.79 (1H, dd, ^3^*J* = 8 Hz, ^4^*J =* 0.6 Hz, 9-H,), 6.92 (1H, td, ^3^*J* = 7.5 Hz, ^4^*J* = 0.6 Hz, 7-H), 7.11 (1H, dd, ^3^*J* = 7.5 Hz, ^4^*J* = 1.4 Hz, 6-H), 7.17 (1H, td, ^3^*J* = 8 Hz, ^4^*J* = 1.4 Hz, 8-H), 7.25 (1H, d, ^4^*J* = 2.4 Hz, 4-H), 7.33 (1H, dd, ^3^*J* = 8.6 Hz, ^4^*J* = 2.4 Hz, 2-H), 8.63 (1H, brs, NH). ^13^C-NMR (100 MHz; DMSO-*d*_6_): δ_C_ 30.3 (3C), 35.4, 45.3, 113.8, 114.2, 119.7, 122.7, 123.7, 123.9, 124.4, 127.2, 127.7, 133.7, 144.6, 145.4, 213.6. HRMS (ESI) *m*/*z*: Calcd. for C_18_H_21_N_2_S_2_ [M + H]^+^ 329.1141; Found 329.1137.

*N-(10-Methyl-10H-phenothiazin-3-yl)benzothioamide* (**2b**). Purification by flash chromatography gave **2b** (0.9 g, 70%) as yellow solid, m.p. 172–174 °C. ^1^H-NMR (400 MHz, DMSO-*d*_6_, Me_4_Si): δ_H_ 3.38 (3H, s, NCH_3_), 6.82 (1H, d, ^3^*J* = 7.8 Hz, 9-H), 6.83 (1H, d, ^3^*J* = 8.5 Hz, 1-H), 6.94 (1H, t, ^3^*J* = 7.3 Hz, 7-H), 7.14 (1H, d, ^3^*J* = 7.3 Hz, 6-H), 7.18 (1H, t, ^3^*J* = 7.8 Hz, 8-H), 7.4–7.44 (2H, m, Ph), 7.48–7.51 (2H, m, Ph, 4-H), 7.58 (1H, dd, ^3^*J* = 8.5 Hz, ^4^*J* = 1.8 Hz, 2-H), 7.83 (2H, d, ^3^*J* = 7.3 Hz, Ph), 8.87 (1H, brs, NH). ^13^C-NMR (100 MHz; DMSO-*d*_6_): δ_C_ 35.5, 113.8, 114.1, 122.6, 122.70, 122.76, 123.2, 124.1, 126.6 (2C), 127.2, 127.6, 128.6 (2C), 131.2, 133.8, 142.9, 144.7, 145.4, 195.1. HRMS (ESI) *m*/*z*: Calcd. for C_20_H_17_N_2_S_2_ [M + H]^+^ 349.0828; Found 349.0831.

*N-(10-Methyl-10H-phenothiazin-3-yl)-4-bromobenzothio-amide* (**2c**). Purification by flash chromatography gave **2c** (0.5 g, 44%) as yellow solid, m.p. 148–150 °C. ^1^H-NMR (400 MHz, DMSO-*d*_6_, Me_4_Si): δ_H_ 3.33 (3H, s, NCH_3_), 6.95–7.02 (3H, m, 9-H, 1-H, 7-H), 7.17 (1H, dd, ^3^*J* = 7.8 Hz, ^4^*J* = 1.3 Hz, 6-H), 7.23 (1H, td, ^3^*J* = 8.0 Hz, ^4^*J* = 1.3 Hz, 8-H), 7.37–7.39 (3H, m, Ph, 2-H, 4-H), 7.75 (2H, d, ^3^*J* = 8.1 Hz, Ph), 11.74 (1H, brs, NH). ^13^C-NMR (100 MHz; DMSO-*d*_6_): δ_C_ 35.7, 114.4, 114.9, 116.7, 121.4, 121.9, 122.2, 123.1, 124.2, 124.8, 127.3, 129.7 (2C), 130.8 (2C), 135.5, 141.8, 143.8, 145.5, 195.8. HRMS (ESI): Calcd. for C_20_H_16_BrN_2_S_2_ [M + H]^+^ 426.9932/428.9912; Found 426.9947/ 428.9923.

*N-(10-Methyl-10H-phenothiazin-3-yl)-4-nitrobenzothioamide* (**2d**). Purification by flash chromatography gave **2d** (1.6 g, 90%) as brown solid, m.p. 165–167°C. ^1^H-NMR (400 MHz, DMSO-*d*_6_, Me_4_Si): δ_H_ 3.34 (3H, s, NCH_3_), 6.96–7.03 (3H, m, 1-H, 9-H, 7-H), 7.18 (1H, dd, ^3^*J* = 8.0 Hz, ^4^*J* = 1.5 Hz, 6-H), 7.24 (1H, td, ^3^*J* = 7.9 Hz, ^4^*J* = 1.5 Hz, 8-H), 7.67 (1H, dd, ^3^*J* = 8.7 Hz, ^4^*J* = 2.4 Hz, 2-H), 7.74 (1H, d, ^4^*J* = 2.4 Hz, 4-H), 7.98 (2H, d, ^3^*J* = 8.8 Hz, Ph), 8.30 (2H, d, ^3^*J* = 8.8 Hz, Ph), 12.02 (1H, brs, NH). ^13^C-NMR (100 MHz; DMSO-*d*_6_): δ_C_ 35.7, 114.7, 115.2, 121.8, 122.3, 122.4, 123.1, 123.7 (2C), 123.8, 127.3, 128.4, 129.1 (2C), 134.9, 144.0, 145.4, 147.8, 148.2, 194.6. HRMS (ESI) *m*/*z*: Calcd. for C_20_H_16_N_3_O_2_S_2_ [M + H]^+^ 394.0678; Found 394.0659.

*N-(10-Methyl-10H-phenothiazin-3-yl)naphthalene-1-carbothioamide* (**2e**). Purification by flash chromatography gave **2e** (1.12 g, 82%) as yellow solid, m.p. 130–133°C. ^1^H-NMR (600 MHz, CDCl_3_, Me_4_Si): δ_H_ 3.40 (3H, s, NCH_3_), 6.84 (2H, d, ^3^*J* = 8.5 Hz, 1-H, 9-H), 6.98 (1H, t, ^3^*J* = 7.5 Hz, 7-H_7_), 7.16 (1H, dd, ^3^*J* = 7.5 Hz, ^4^*J* = 1.2 Hz, 6-H), 7.21 (1H, td, ^3^*J* = 8.5 Hz, ^4^*J* = 1.2 Hz, 8-H), 7.50–7.56 (3H, m, Naph), 7.62 (1H, d, ^4^*J* = 2.3 Hz, 4-H), 7.65 (1H, d, ^3^*J* = 7.0 Hz, Naph), 7.69 (1H, dd, ^3^*J* = 8.5 Hz, ^4^*J* = 2.3 Hz, 2-H), 7.89 (2H, d, ^3^*J* = 8.2 Hz, Naph), 8.2 (1H, d, ^3^*J* = 8.2 Hz, Naph), 8.96 (1H, brs, NH). ^13^C-NMR (125 MHz, CDCl_3_): δ_C_ 35.7, 114.7, 115.1, 121.8, 122.0, 122.3, 123.0, 123.2, 124.3, 125.4, 125.7, 126.6, 127.1, 127.3, 128.4, 128.5, 128.7, 129.2, 133.5, 136.1, 143.3, 143.5, 145.6, 196.4. HRMS (ESI) *m*/*z*: Calcd. for C_24_H_19_N_2_S_2_ [M + H]^+^ 399.0984; Found 399.0997.

### 4.3. General Procedure for Obtaining Thiazolo[5,4-b]phenothiazine *(**3a**–**e**)*

The previously reported procedure for benzothiazole synthesis by iron catalysed oxidative C-S bond formation [18] was adapted. Thus, a mixture of thioamide, potassium persulfate (K_2_S_2_O_8_), 2 equivalents of pyridine, and 10 mol % FeCl_3_ in dimethylsulfoxide DMSO (20 mL) was stirred at 80 °C for 4 h. The reaction mixture was cooled down to room temperature, and diluted with water. After extraction with ethyl acetate (3 × 25 mL), the combined organic layers were washed with brine (20 mL), and dried over anh. MgSO_4_. The solvent was evaporated and the residue was purified by column chromatography (silica gel, toluene, or toluene/ethylacetate = 20:1) which gave **3a**–**e** as yellowish solids which were recrystallized from ethanol.

*2-(t-Butyl)-10-methyl-10H-thiazolo[5,4-b]phenothiazine* (**3a**). Thioamide **2a** gave **3a** (0.338 g, 26%) as grey solid, m.p. 183–185 °C. ^1^H-NMR(400 MHz, CDCl_3_, Me_4_Si): δ_H_ 1.48 (9H, s, *tBu*), 3.41 (3H, s, NCH_3_), 6.83 (1H, dd, ^3^*J* = 8.2Hz, ^4^*J* = 0.6 Hz, 9-H), 6.95 (1H, td, ^3^*J* = 7.6 Hz, ^4^*J* = 1.0 Hz, 7-H), 7.16–7.2 (3H, m, 6-H, 8-H, 11-H), 7.74 (1H, s, 4-H). ^13^C-NMR (100 MHz, CDCl_3_): δ_C_ 30.8 (3C), 36.0, 38.3, 105.7, 114.5, 120.4, 122.6, 123.4, 123.7, 127.2, 127.6, 134.9, 143.6, 145.6, 149.2, 180.0. IR (KBr, ν, cm^−1^) 3030 (w), 2961(m), 1574 (m), 1506 (m), 1470 (m), 1321 (m), 1278 (m), 870 (s), 846 (s), 753 (s). HRMS (ESI) *m*/*z*: Calcd. for C_18_H_19_N_2_S_2_ [M + H]^+^ 327.0984; Found 327.0995.

*2-Phenyl-10-methyl-10H-thiazolo[5,4-b]phenothiazine* (**3b**). Thioamide **2b** gave **3b** (0.386 g, 44%) as pale-yellow solid, m.p. 163–165 °C. ^1^H-NMR (400 MHz, CDCl_3_, Me_3_Si): δ_H_ 3.42 (3H, s, NCH_3_), 6.84 (1H, d, ^3^*J* = 7.8 Hz, 9-H), 6.97 (1H, td, ^3^*J* = 8.2 Hz, ^4^*J* = 0.76 Hz, 7-H), 7.17–7.21 (3H, m, 6-H, 8-H, 11-H), 7.45–7.47 (3H, m, Ph), 7.8 (1H, s, 4-H), 8.01 (2H, m, Ph). ^13^C-NMR (100 MHz, CDCl_3_): δ_C_ 36.0, 105.7, 114.6, 120.8, 122.8, 123.4, 124.2, 127.30, 127.32 (2C), 127.7, 129 (2C), 130.7, 133.7, 135.1, 144.1, 145.4, 150.2, 165.5. IR (KBr, ν_,_ cm^−1^) 3020 (w), 2961 (m), 1571 (m), 1506 (m), 1478 (m), 1326 (m), 1284 (m), 761 (s), 744 (s). HRMS (ESI) *m*/*z*: Calcd. for C_20_H_15_N_2_S_2_ [M + H]^+^ 347.0671; Found 347.0678.

*2-(4-Bromophenyl)-10-methyl-10H-thiazolo[5,4-b]-phenothiazine* (**3c**). Thioamide **2c** gave **3c** (0.13 g, 26%) as pale-yellow solid, m.p. 176–178 °C. ^1^H-NMR (400 MHz, CDCl_3_, Me_3_Si): δ_H_ 3.44 (3H, s, NCH_3_), 6.85 (1H, dd, ^3^*J* = 7.7 Hz, ^4^*J* = 0.9 Hz, 9-H), 6.97 (1H, td, ^3^*J* = 7.6 Hz, ^4^*J* = 0.9 Hz, 7-H), 7.18–7.22 (3H, m, 6-H, 8-H, 11-H), 7.58 (2H, d, ^3^*J* = 8.5 Hz, Ph), 7.78 (1H, s, 4-H), 7.87–7.89 (2H, d, ^3^*J* = 8.5 Hz, Ph). ^13^C-NMR (100 MHz, CDCl_3_): δ_C_ 36.1, 105.7, 114.7, 120.9, 122.9, 123.4, 124.5, 125, 127.3, 127.7, 128.7 (2C), 132.29 (2C), 132.5, 135.2, 144.4, 145.3, 150.1, 164.6. IR (KBr, ν, cm^−1^) 3059 (w), 2962 (m), 1558 (m), 1505 (m), 1471 (m), 1327 (m), 1265 (m), 942 (s), 868 (s), 825 (s), 753 (s). HRMS (ESI) *m*/*z*: Calcd. for C_20_H_14_BrN_2_S_2_ [M + H]^+^ 424.9776/426.9756, Found 424.9791/426.9768.

*2-(4-Nitrophenyl)-10-methyl-10H-thiazolo[5,4-b]-phenothiazine* (**3d**). Thioamide **2d** gave **3d** (0.6 g, 51%) as pale-yellow solid, m.p. 206–208 °C. ^1^H-NMR (400 MHz, CDCl_3_, Me_3_Si): δ_H_ 3.47 (3H,s, NCH_3_), 6.88 (1H, d, ^3^*J* = 8.0 Hz, 9-H), 6.99 (1H, t, ^3^*J* = 7.1 Hz, 7-H), 7.16–7.26 (3H, m, 6-H, 8-H, 11-H), 7.84 (1H, s, 4-H_4_), 8.16 (2H, d, ^3^*J* = 8.8 Hz, Ph), 8.30 (2H, d, ^3^*J* = 8.8 Hz, Ph). ^13^C-NMR (100 MHz, CDCl_3_): δ_C_ 36.2, 105.5, 114.8, 121.3, 123.1, 123.2, 124.4 (2C), 125.3, 127.3, 127.9 (2C), 128.3, 129.1, 135.9, 139.3, 145.1, 148.7, 150.2, 162.5. IR (KBr, ν, cm^−1^) 3059 (w), 2920 (m), 1595 (m), 1502 (s), 1466 (m), 1324 (s), 1291 (m), 871 (s), 851 (s), 829 (s), 749 (s). HRMS (ESI) *m*/*z*: Calcd. for C_20_H_14_N_3_S_3_ [M + H]^+^ 392.0522; Found 392.0529.

*2-(Naphthalen-1-yl)-10-methyl-10H-thiazolo[5,4-b]-phenothiazine* (**3e**). Thioamide **2e** gave **3e** (0.24 g, 32%) as pale-yellow solid, m.p. 204–206 °C. ^1^H-NMR (600 MHz, CDCl_3_, Me_3_Si): δ_H_ 3.46 (3H, s, NCH_3_), 6.87 (1H, d, ^3^*J* = 7.8 Hz, 9-H), 6.98 (1H, t, ^3^*J* = 7.4 Hz, 7-H), 7.20–7.23 (2H, m, 6-H, 8-H), 7.28 (1H, s, 11-H), 7.52–7.57 (2H, m, Naph), 7.61 (1H, td, ^3^*J* = 8.4 Hz, ^4^*J* = 1.0 Hz, Naph), 7.88 (1H, d, ^3^*J* = 7.0 Hz, Naph), 7.91 (1H, d, ^3^*J* = 8.4 Hz, Naph), 7.93 (1H, s, 4-H), 7.96 (1H, d, ^3^*J* = 8.3 Hz, Naph), 8.96 (1H, d, ^3^*J* = 8.4 Hz, Naph). ^13^C-NMR (125 MHz, CDCl_3_): δ_C_ 36.1, 105.4, 114.6, 121.1, 122.9, 123.5, 124.3, 125.1, 126.0, 126.6, 127.3, 127.73, 127.75, 128.5, 129.3, 130.7, 130.8, 131.0, 134.1, 135.5, 144.3, 145.5, 150.3, 165.6. IR (KBr, ν, cm^−1^) 3059 (w), 2962 (m), 1574 (m), 1472 (m), 1440 (m), 1265 (m), 1141 (m), 925 (s), 876 (s), 829 (s), 750 (s); HRMS (ESI) *m*/*z*: Calcd. for C_24_H_17_N_2_S_2_ [M + H]^+^ 397.0828; Found 397.0834.

### 4.4. Biologic Assay

Instrumentation for in vitro testing: Lamil Plus class II Laminary Hoods (from Karstulan Metalli Oy, Karstula, Finland), Uniequip incubator with CO_2_ (Martinsried, Germany), Synergy II microplate reader from BioTek Instruments (Winooski, VT, USA), Universal 32R centrifuge with swing-out rotor (from Hettich, Tuttlingen, Germany), Titramax 1000 incubator with shaker (from Heidolph Instruments, Schwabach, Germany), BX40 optical microscope and CKX41 inverted phase fluorescence microscope (from Olympus Corporation, Center Valley, PA, USA).

#### 4.4.1. Cell Cultures

HL-60 human promyelocytic- and THP-1 human monocytic leukaemia cell lines were acquired from European Cell Culture Collection. Cells were cultivated in RPMI-1640 media supplemented with 10% fetal bovine serum, 2 mM glutamine, and 1% 2-[4-(2-hydroxyethyl)piperazin-1-yl]etansulfonic acid HEPES buffer (all reagents from Sigma Aldrich, St. Louis, MO, USA). Human peripheral blood mononuclear cells (PBMCs) were isolated from whole blood, obtained by venepuncture from a 43-year-old female donor, following her informed written consent. PBMCs were isolated in density gradient, using Histopaque 1077 separation media and Hanks Modified Eagle’s Media (both from Sigma Aldrich) for cell wash, using our previously described method [36].

For colorimetric testing, cells were seeded on 96-well micro plates, at a concentration of 3 × 10^4^ cells/100 μL media, and incubated for 24 h (Nunclon Delta surface plates from Thermo Fischer Scientific, Waltham, MA, USA). For fluorescence measurements, black, clear-bottom 96-well plates were used (from Corning, Tewksbury, MA, USA).

#### 4.4.2. Cell Viability Tests

Cell viability was tested with Trypan Blue staining and optical microscope examination. Stock solutions of 40 mM in dimethyl sulfoxide (DMSO, from Titolchimica, Pontevecchio Polesine, RO, Italy) are prepared. Compounds were diluted in a mixture of DMSO:PEG 400 in a proportion of 1:1. Twenty micromolar (20 mM) solutions of tested compounds were prepared in a sterile glucose 5% solution (Braun Melsungen AG, Melsungen, Germany), slightly acidified to pH 6.8–6.9 to obtain a better solubility; the solutions were sonicated. Cells were treated with the tested compounds at dilution series of 1000 to 1.25 μM final concentrations in cell suspension. As positive reference, 0.01–250 μM solutions of cytarabine (Cytosar, from Actavis, Dublin, Ireland)—an antineoplastic agent used in leukaemia, which inhibits the synthesis of deoxyribonucleic acid—were used.

The compounds potential for auto-fluorescence and colour background was tested. The cytotoxicity of the compounds was assessed using the MTT colorimetric method [37]; the IC_50_ parameter was calculated from the dose-response curve (GraphPad Prism 5 software, version 5, GraphPad Company, San Diego, CA, USA).

The metabolic activity of the treated cells was evaluated using the resazurin-based quantitative method (Alamar Blue reagent purchased from Life Technologies, Carlsbad, CA, USA), as an alternative to cell viability testing. The cytoplasm of viable cells is able to reduce resazurin to resorufin, a red-colour and highly fluorescent dye, whose intensity is related to the number of living cells within the population. The cells were plated on 96-well microplates and treated in the same way and with identical concentrations as for the MTT test, the wells were stained with Alamar Blue in a proportion of 20:100 μL, and after 2 h of incubation the fluorescence emission was measured at 620 nm.

Apoptosis was measured using Alexa Fluor 480-labelled AnexinV marker, and with the propidium iodide stain which binds the DNA by random intercalation between nuclear bases in death cells (reagents from Life Technologies). The cells were seeded in 24-well plates at a concentration of 10^6^ cells/mL media and treated for 4 and 8 h, respectively, with a final concentration of 250 μM tested compound. The harvested cells were washed in cold PBS and suspended in staining buffer. Samples were divided in two aliquots, stained with Alexa Fluor Annexin V, or with PI, respectively. After 15 min incubation, the cells were washed in cold PBS, suspended in 100 μL buffer, and the fluorescence was measured in triplicate at 530–575 nm using 488 nm excitation.

## Figures and Tables

**Table 1:** Experimental UVultraviolet-vis spectral properties and computational data for TAPTZ derivatives.

Cpd	λ_max,abs_^1^ (nm) (ε (M^−1^ cm^−1^))	λ_max, em_^2^ (nm)	Stokes Shift ^3^ (cm^−1^)	E_gap,opt_^4^ (eV)	E_HOMO_^5^ (eV)	E_LUMO_^5^ (eV)	Dipole Moment ^5^ (D)
**3a**	263 (28,900) 326 (7400)	447	8300	4.49	−5.07	−0.78	2.68
**3b**	261 (43,400) 370 (15,800)	550	8800	4.28	−5.07	−1.54	2.67
**3c**	262 (32,800) 377 (12,200)	564	8800	4.18	−5.17	−1.77	3.92
**3d**	264 (29,600) 300 (12,600) 424 (9300)	-	-		−5.36	−2.72	7.63
**3e**	250 (23,300) 294 (8900) 373 (8900)	608	10,400	4.07	−5.07	−1.69	2.55

^1^ 10^−5^ M in acetonitrile solution; ^2^ after irradiation with the longest absorption wavelength; ^3^ Δν = 1/λ_max,abs_ − 1/λ_max,e_; ^4^ determined from the cross-section of absorption and emission spectra; ^5^ computed using density functional theory (DFT) level of theory B3LYP hybrid functional with 6-31G* basis set.

**Table 2:** Half inhibitory concentration (IC_50_) calculated using sigmoidal dose-response relationship for *N*-(phenothiazine-3-yl)-thioamides (**2a**, **2c**, **2e**), TAPTZ **3a**, **3c**, **3e**, and cytarabine, respectively, against THP-1 and HL-60 tumour cells and normal peripheral blood mononuclear cells (PBMCs) in vitro.

Cpd	Antiproliferative Activity IC_50_ (μM)		
	THP-1	HL-60	PBMC
**2a**	101.70 ± 0.11 ^b^	175.8 ± 0.23 ^b^	>2000
**2b**	1481 ^a^	>2000	1121
**2c**	88.9 ± 0.03 ^b^	69.1 ± 0.19 ^b^	>2000
**2e**	>2000	>2000	227 ^a^
**3a**	463.4 ± 0.16 ^b^	194.3 ± 0.17 ^b^	>2000
**3b**	1241 ^a^	338.9 ± 0.14 ^b^	1906 ^a^
**3c**	>1000	1066 ^a^	>1000
**3e**	21.6 ± 0.06 ^b^	67.2 ± 0.09 ^b^	>2000
Cytarabine	9.0 ± 0.05 ^b^	13.5 ± 0.05 ^b^	

^a^ Estimated outside the 95% confidence interval; ^b^ Standard deviation of three time independent tests.

## Supplementary Materials

Supplementary materials can be found at www.mdpi.com/1422-0067/18/7/1365/s1.
