# Supplementary material for: Novel Thiazolo[5,4-b]phenothiazine Derivatives: Synthesis, Structural Characterization, and In Vitro Evaluation of Antiproliferative Activity against Human Leukaemia
Source: Int J Mol Sci. 2017 Jun 26;18(7):1365. doi: 10.3390/ijms18071365 (PMC5535858; doi:10.3390/ijms18071365)
Supplement: Supplementary file 1 [file ijms-18-01365-s001.zip › ijms-200398-supplementary-final.pdf]

Supplementary material for:

Novel Thiazolo[5,4-b]phenothiazine Derivatives: Synthesis, Structural Characterization, and In Vitro Evaluation of Antiproliferative Activity against Human Leukaemia

B. Brem[1], E. Gal[1], L. Găină[1], L. Silaghi-Dumitrescu[1], E. Fischer-Fodor[2,3], C. I. Tomuleasa[2,4], A. Grozav[5], V. Zaharia[5] L. Filip[5*] and C. Cristea[1]*

Address. [1] Faculty of Chemistry and Chemical Engineering, Babes-Bolyai University, Cluj-Napoca, Romania. [2] Tumor Biology Department, I. Chiricuta Oncology Institute, Cluj Napoca, Romania. [3] Medfuture Research Center, I. Hatieganu University of Medicine and Pharmacy, Cluj Napoca, Romania [4] Research Center for Functional Genomics and Translational Medicine, I. Hatieganu University of Medicine and Pharmacy, Cluj Napoca, Romania.[5] Faculty of Pharmacy, Haţieganu" University of Medicine and Pharmacy, Cluj-Napoca, Romania.

*Corresponding authors

Table of contents

NMR-spectra of 3a-3e **S2-S5**

2D NOESY of 3a **S6**

HRMS spectra of 3a-3e **S7-S9**

Frontier molecular orbital plots of 3a-3d **S10**

In vitro cell metabolic activity in populations of THP-1, HL-60, and PBMC cell cultures **S11**

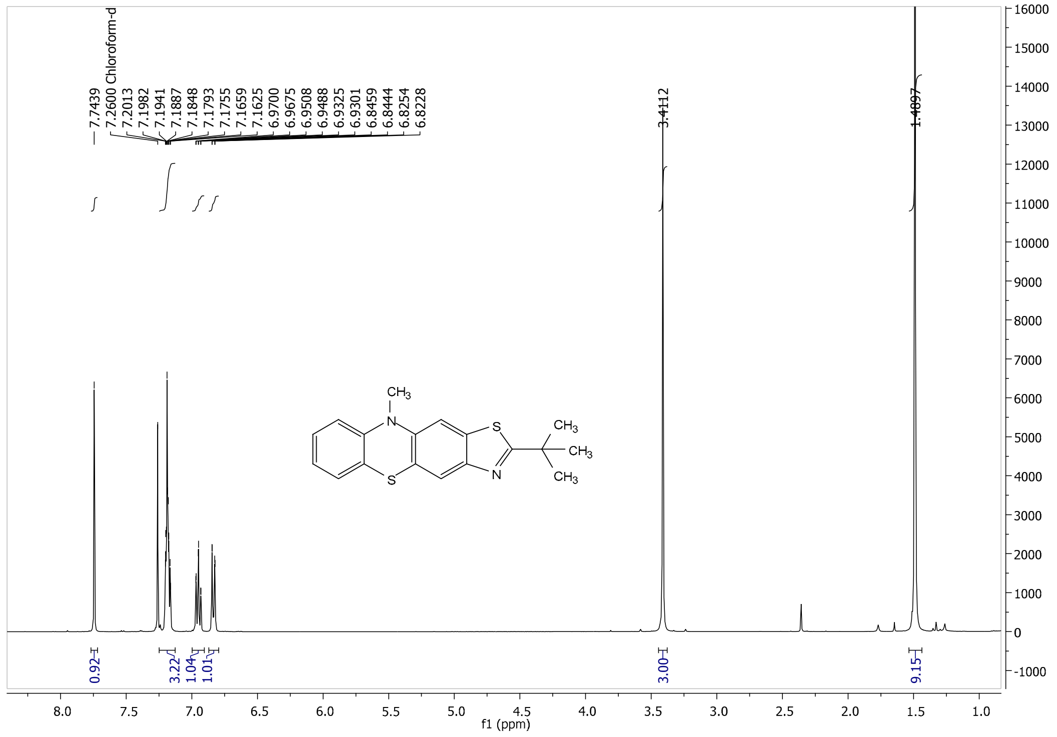

7.7439
7.2600 Chloroform-d
7.2013
7.1982
7.1941
7.1887
7.1848
7.1793
7.1755
7.1659
7.1625
6.9700
6.9675
6.9508
6.9488
6.9325
6.9301
6.8459
6.8444
6.8254
6.8228
3.4112
1.4897
0.92
3.22
1.04
1.01
3.00
9.15
f1 (ppm)

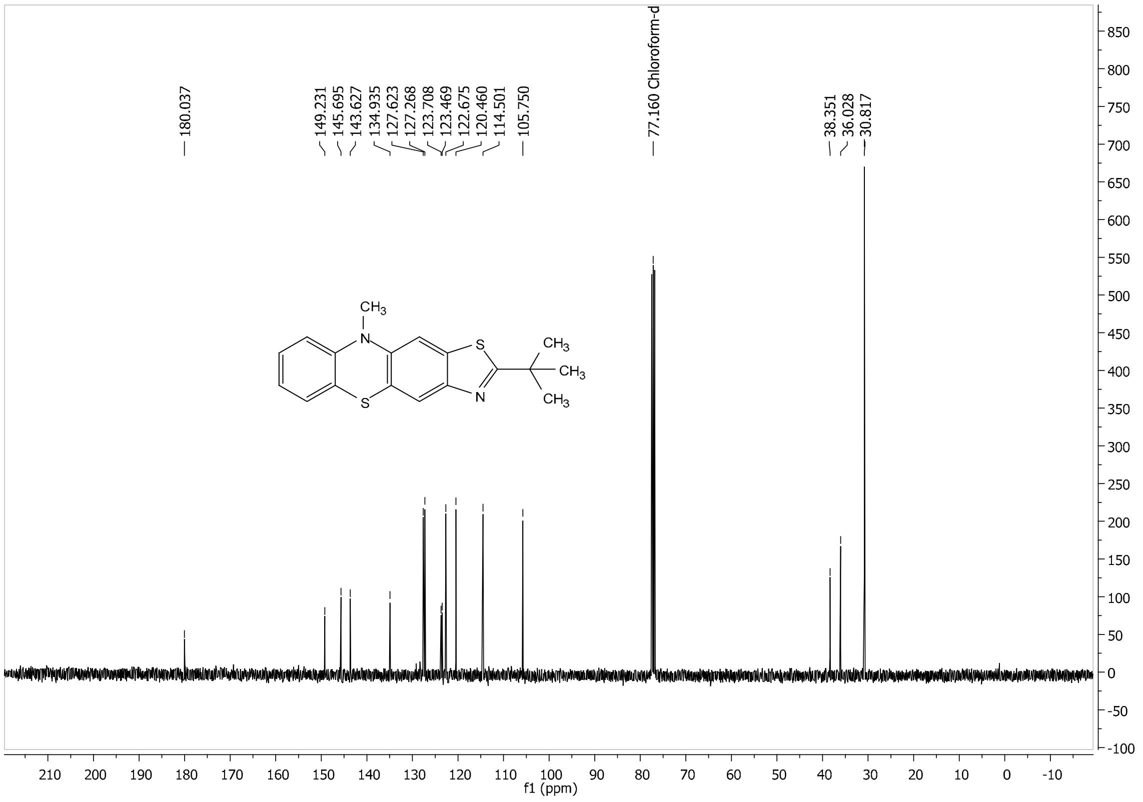

180.037
149.231
145.695
143.627
134.935
127.623
127.268
123.708
123.469
122.675
120.460
114.501
105.750
77.160 Chloroform-d
38.351
36.028
30.817
f1 (ppm)

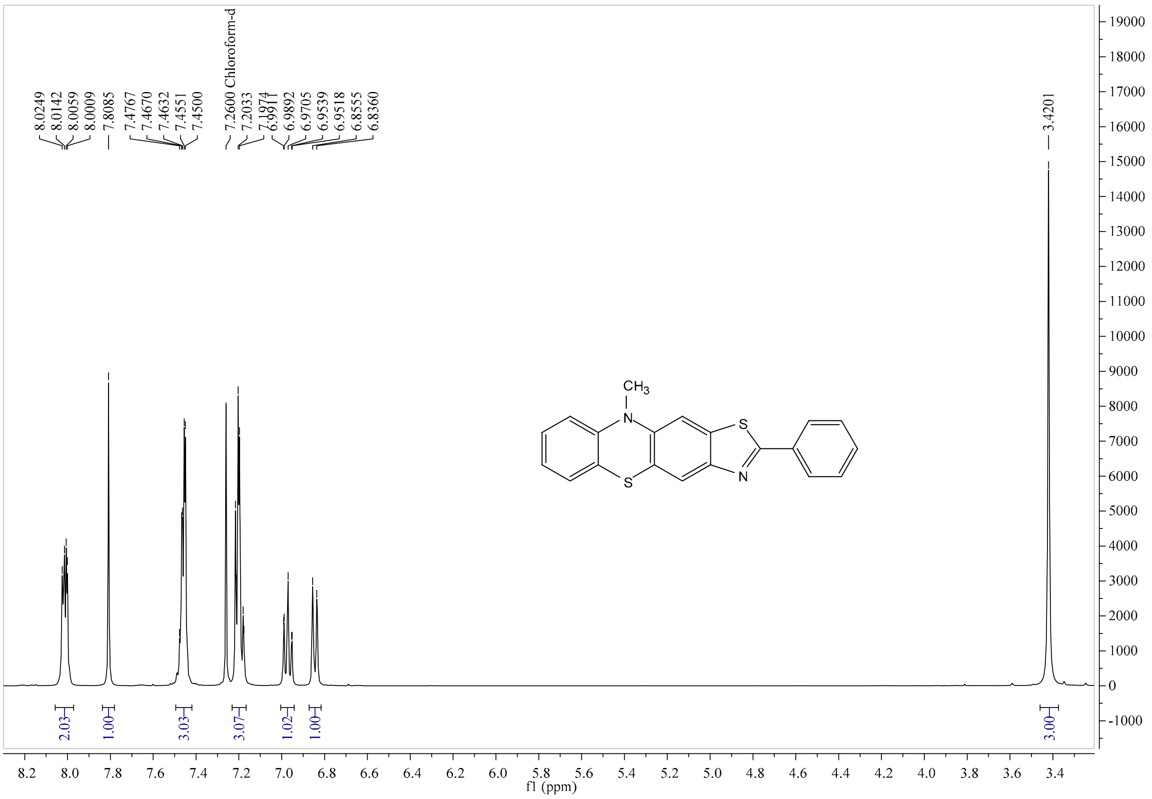
8.0249
8.0142
8.0059
8.0009
7.8085
7.4767
7.4670
7.4632
7.4551
7.4500
7.2600 Chloroform-d
7.2033
6.9892
6.9705
6.9539
6.9518
6.8555
6.8360
3.4201
2.03
1.00
3.03
3.07
1.02
1.00
3.00
CH3
N
S
S
N
f1 (ppm)

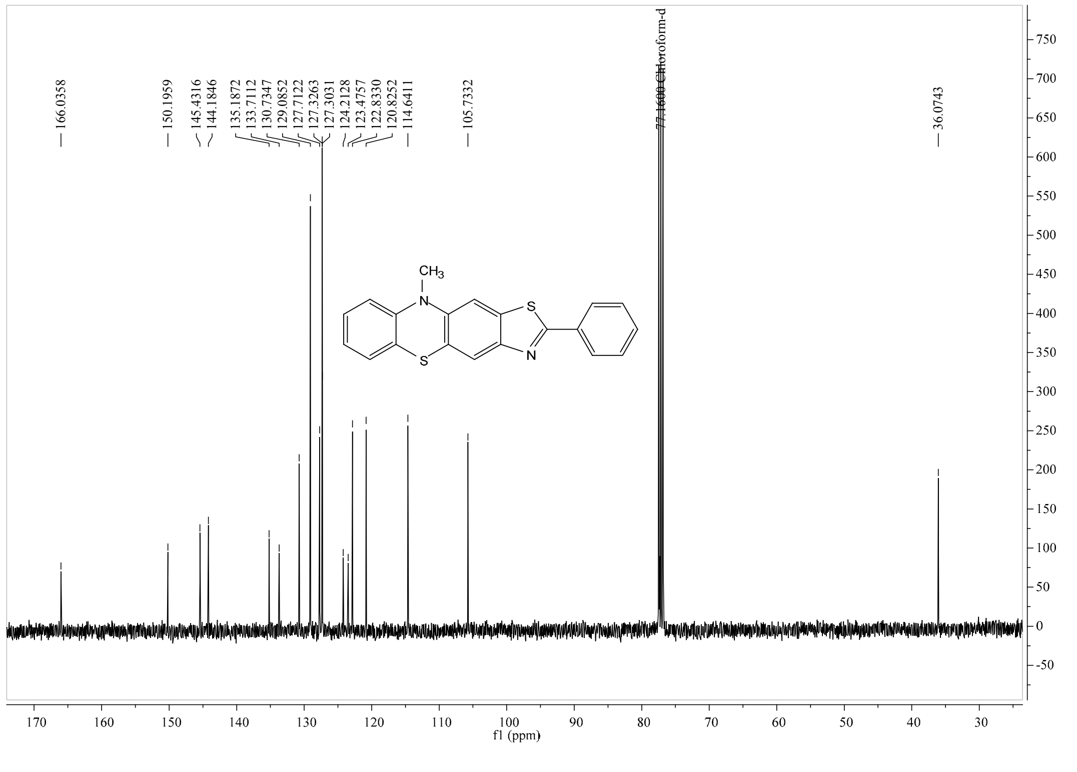
166.0358
150.1959
145.4316
144.1846
135.1872
133.7112
130.7347
129.0852
127.7122
127.3263
127.3031
124.2128
123.4757
122.8330
120.8252
114.6411
105.7332
Chloroform-d
36.0743
CH3
N
S
S
N
f1 (ppm)

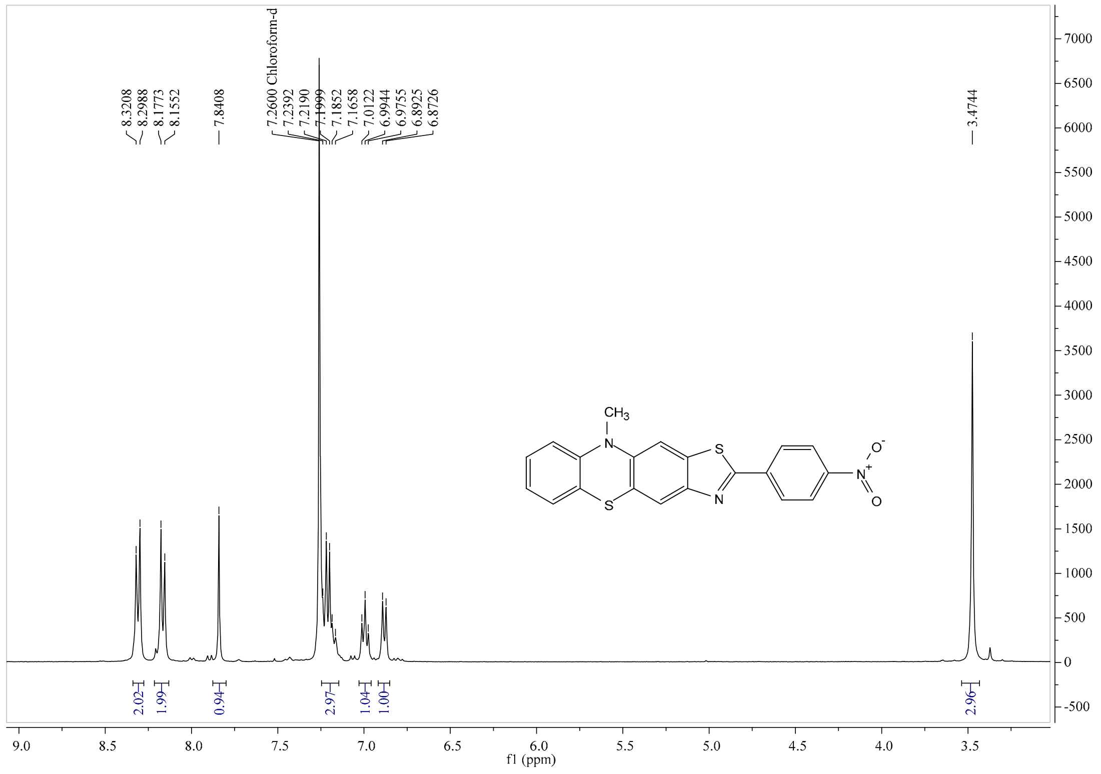
8.3208
8.2988
8.1773
8.1552
7.8408
7.2600 Chloroform-d
7.2392
7.2190
7.1999
7.1852
7.1658
7.0122
6.9944
6.9755
6.8925
6.8726
3.4744
2.02
1.99
0.94
2.97
1.04
1.00
2.96
CH3
N
S
N
S
O-
N+
O
f1 (ppm)

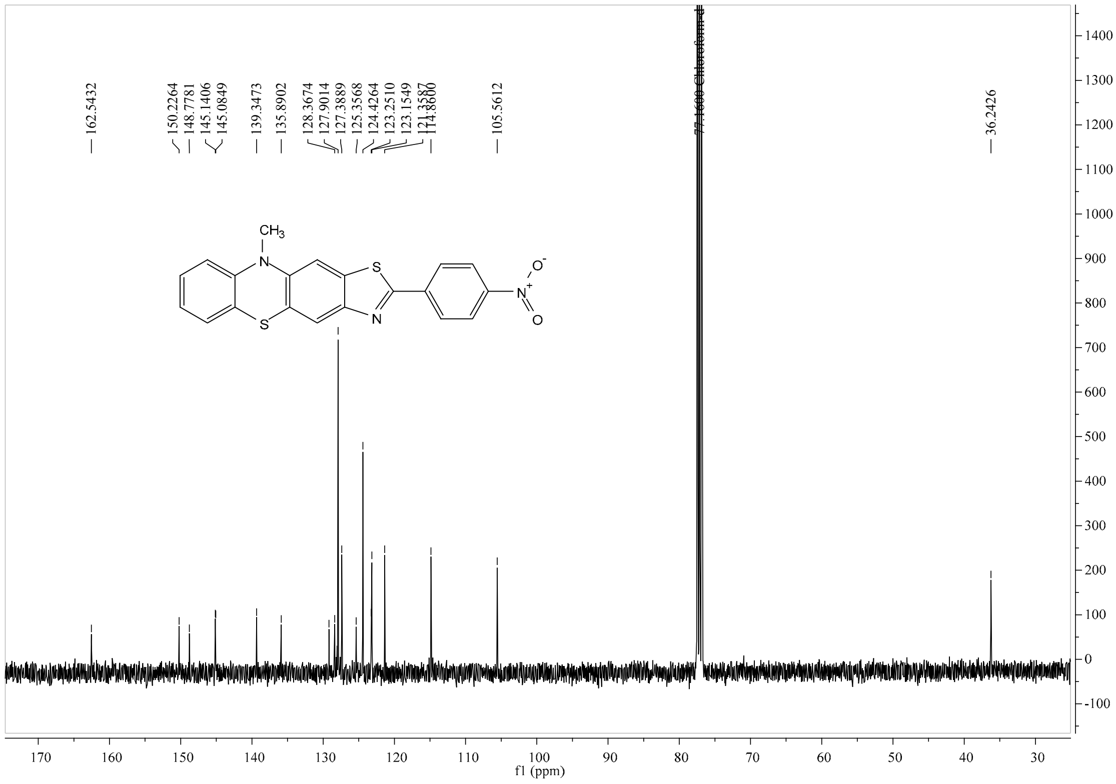
162.5432
150.2264
148.7781
145.1406
145.0849
139.3473
135.8902
128.3674
127.9014
127.3889
125.3568
124.4264
123.2510
123.1549
121.3587
114.8600
105.5612
36.2426
CH3
N
S
N
S
O-
N+
O
f1 (ppm)

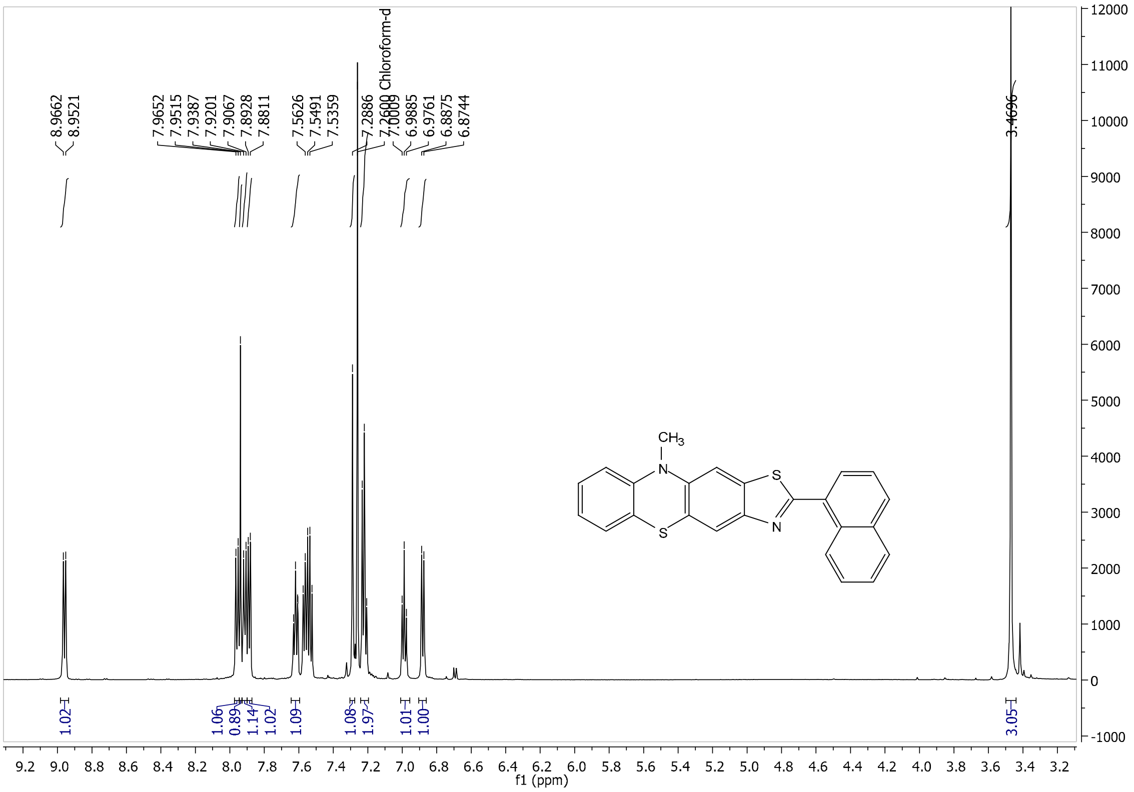

8.9662
8.9521
7.9652
7.9515
7.9387
7.9201
7.9067
7.8928
7.8811
7.5626
7.5491
7.5359
7.2886
7.2600 Chloroform-d
7.0009
6.9885
6.9761
6.8875
6.8744
3.4696
1.02
1.06
0.89
1.14
1.02
1.09
1.08
1.97
1.01
1.00
3.05
CH3
N
S
S
N
f1 (ppm)

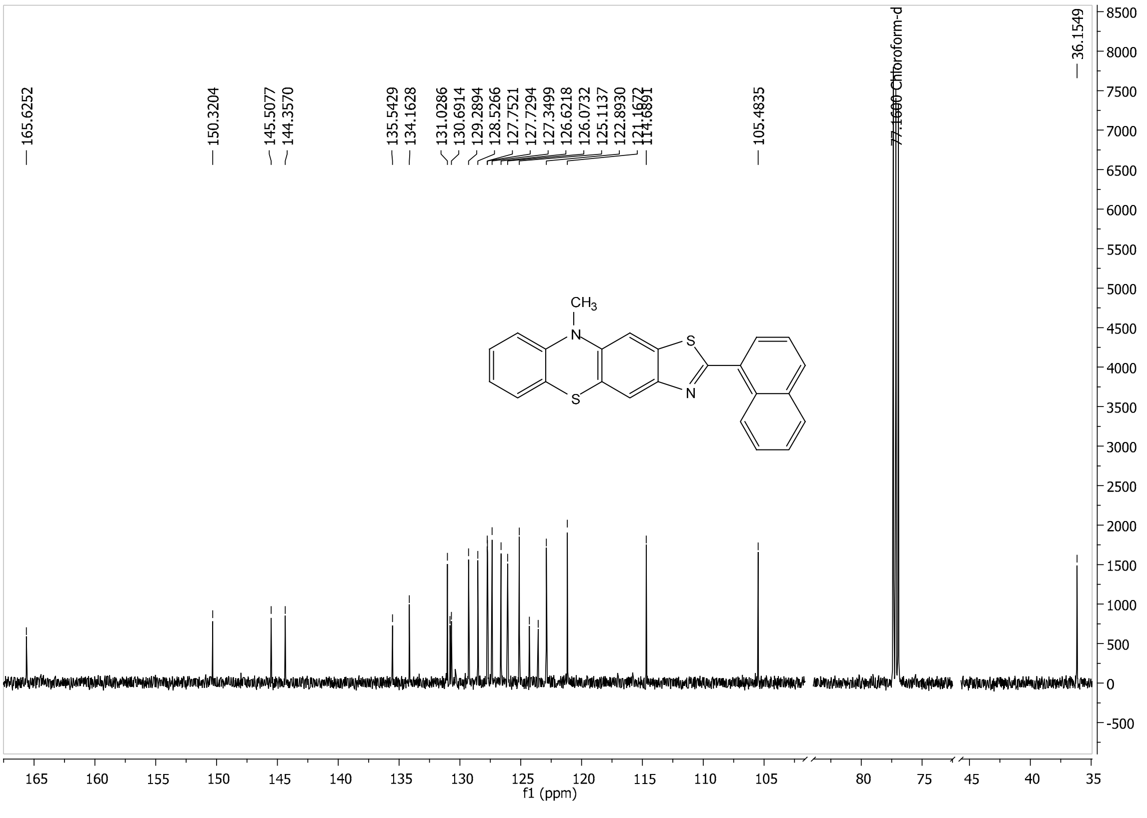

165.6252
150.3204
145.5077
144.3570
135.5429
134.1628
131.0286
130.6914
129.2894
128.5266
127.7521
127.7294
127.3499
126.6218
126.0732
125.1137
122.8930
121.1672
114.6891
105.4835
77.1600 Chloroform-d
36.1549
CH3
N
S
S
N
f1 (ppm)

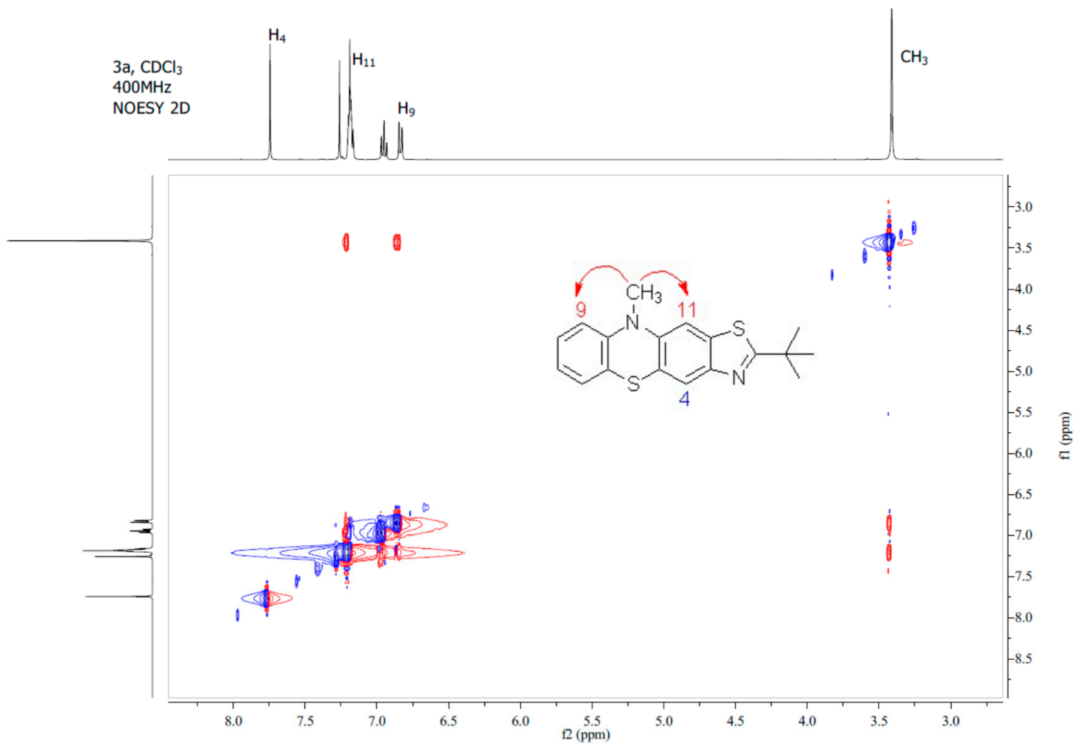

3a, CDCl3
400MHz
NOESY 2D
H4
H11
H9
CH3
CH3
9
11
N
S
S
N
4
f2 (ppm)
f1 (ppm)

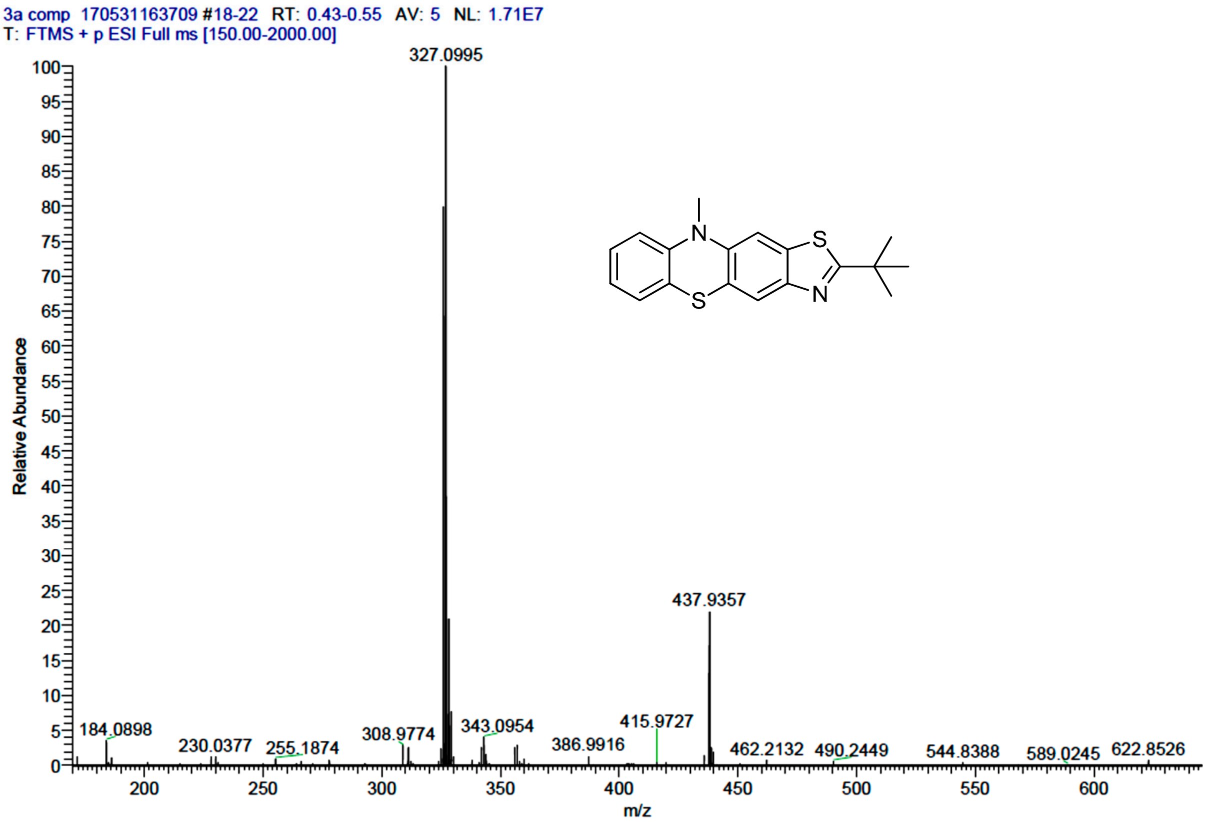
3a comp 170531163709 #18-22 RT: 0.43-0.55 AV: 5 NL: 1.71E7
T: FTMS + p ESI Full ms [150.00-2000.00]
327.0995
437.9357
184.0898
230.0377
255.1874
308.9774
343.0954
386.9916
415.9727
462.2132
490.2449
544.8388
589.0245
622.8526
Relative Abundance
m/z
N
S
S
N

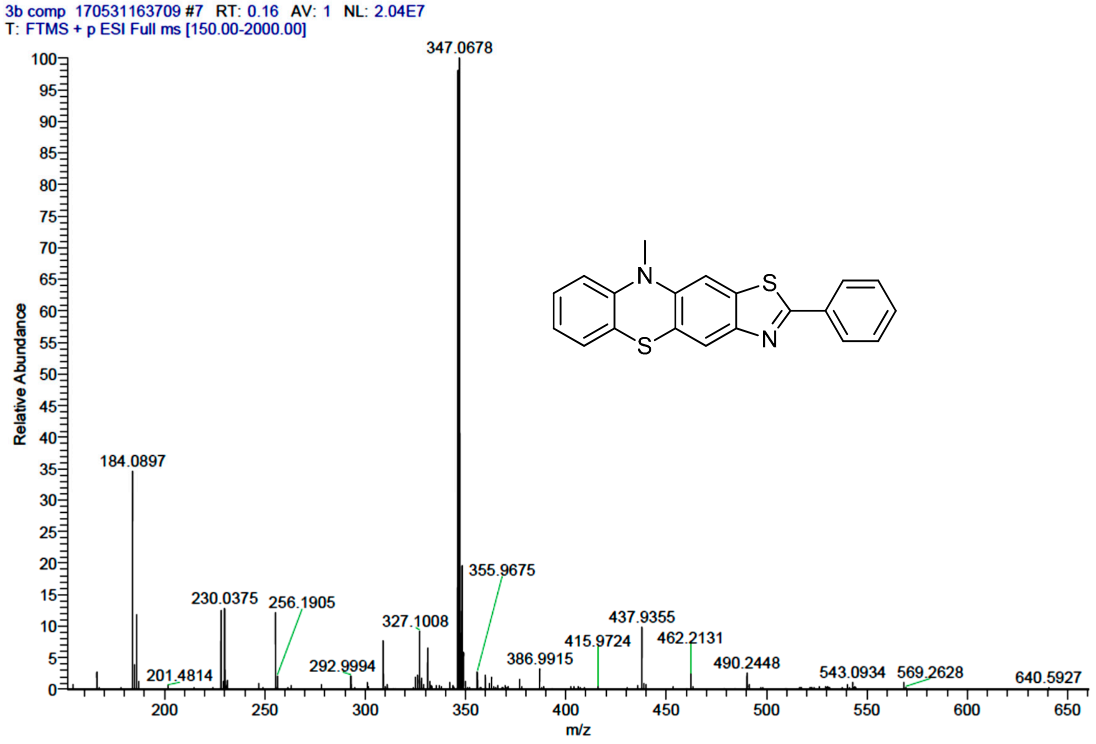
3b comp 170531163709 #7 RT: 0.16 AV: 1 NL: 2.04E7
T: FTMS + p ESI Full ms [150.00-2000.00]
347.0678
184.0897
230.0375
256.1905
201.4814
292.9994
327.1008
355.9675
386.9915
415.9724
437.9355
462.2131
490.2448
543.0934
569.2628
640.5927
Relative Abundance
m/z
N
S
S
N

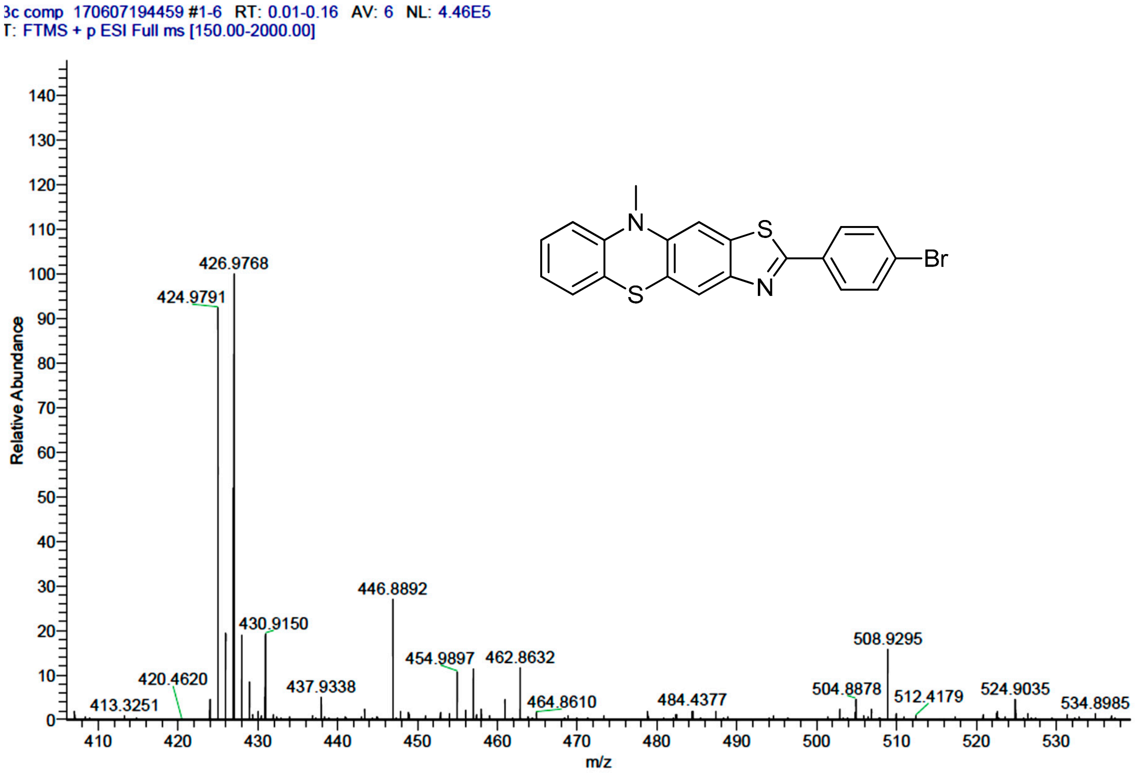

3c comp 170607194459 #1-6 RT: 0.01-0.16 AV: 6 NL: 4.46E5
T: FTMS + p ESI Full ms [150.00-2000.00]
Relative Abundance
m/z
426.9768
424.9791
446.8892
430.9150
508.9295
454.9897
462.8632
420.4620
437.9338
504.8878
524.9035
512.4179
484.4377
464.8610
413.3251
534.8985
N
S
S
N
Br

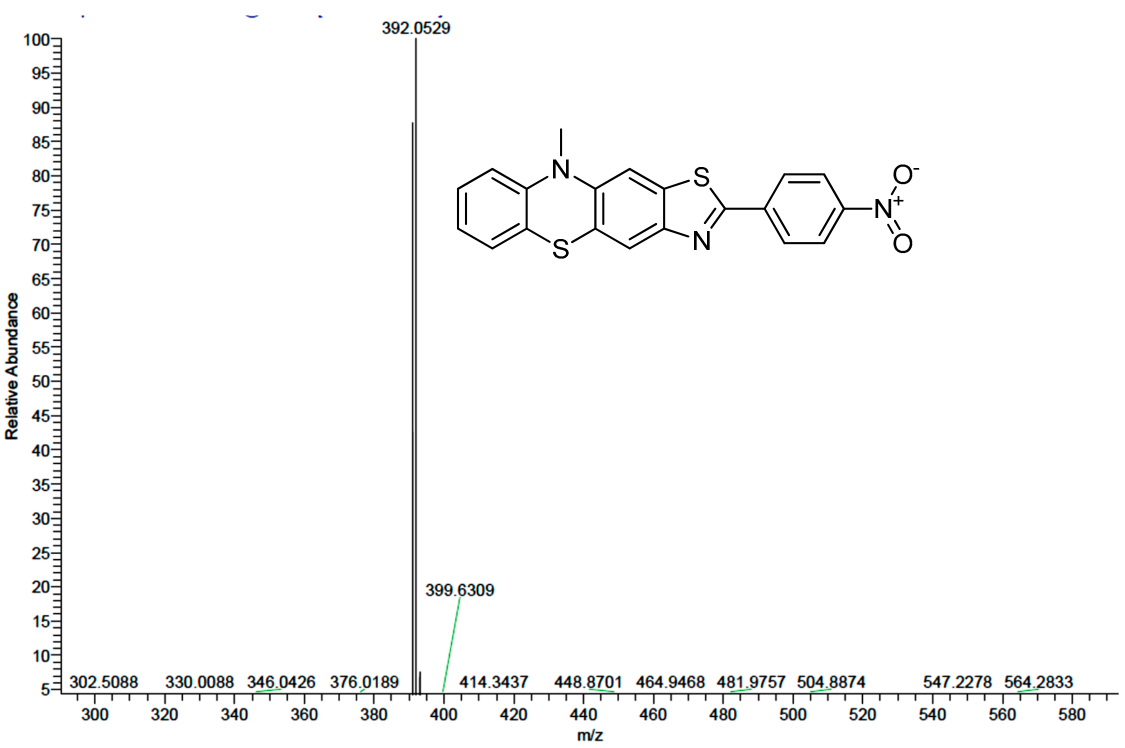
392.0529
399.6309
302.5088
330.0088
346.0426
376.0189
414.3437
448.8701
464.9468
481.9757
504.8874
547.2278
564.2833
Relative Abundance
m/z
N
S
S
N
N+
O-
O

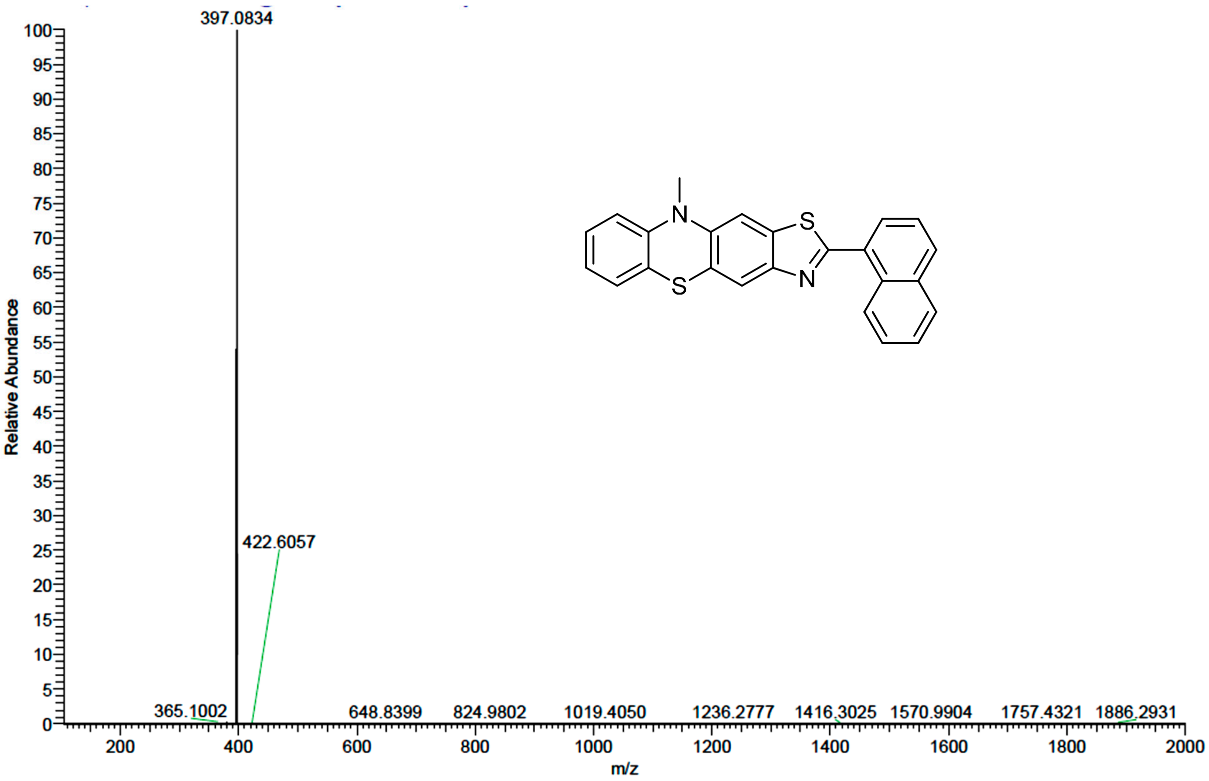

397.0834
422.6057
365.1002
648.8399
824.9802
1019.4050
1236.2777
1416.3025
1570.9904
1757.4321
1886.2931
Relative Abundance
m/z
N
S
S
N

Frontier MO plots

Cpd	HOMO	LUMO
3a	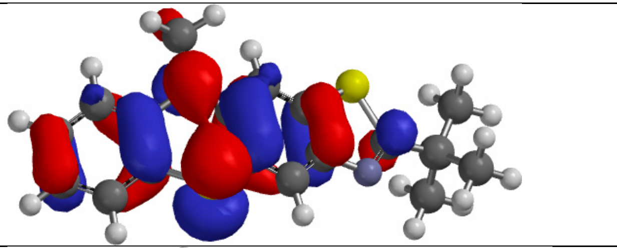	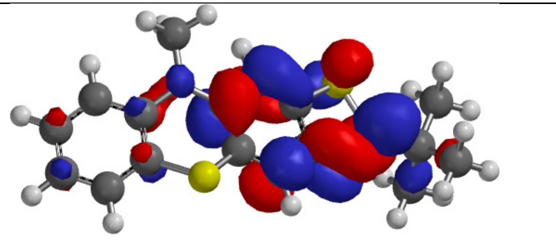
3b	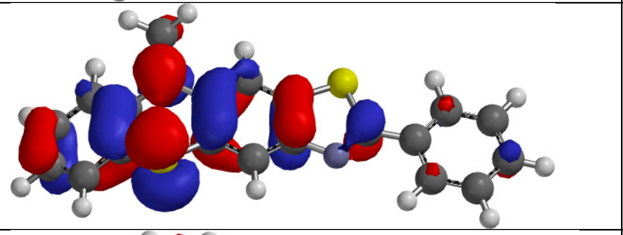	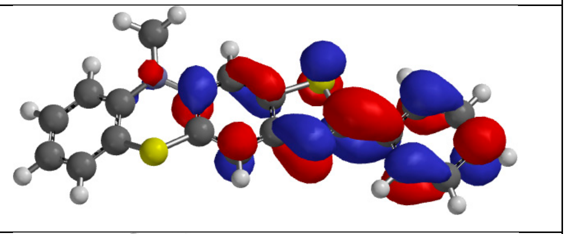
3c	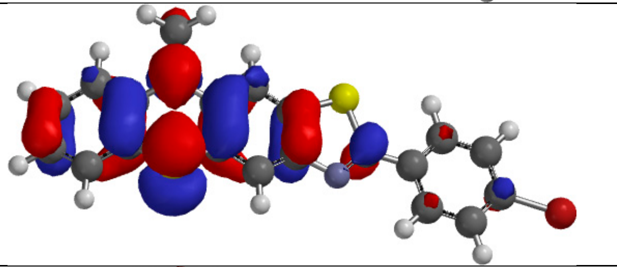	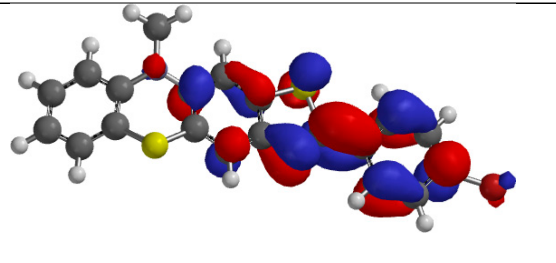
3d	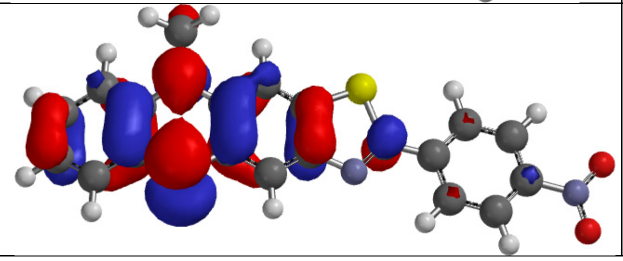	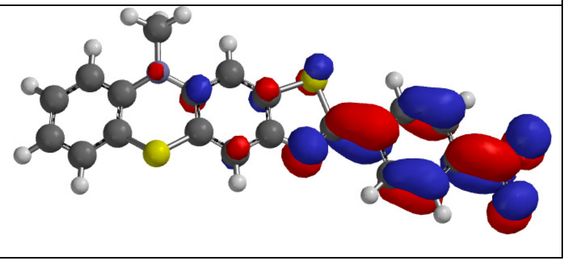

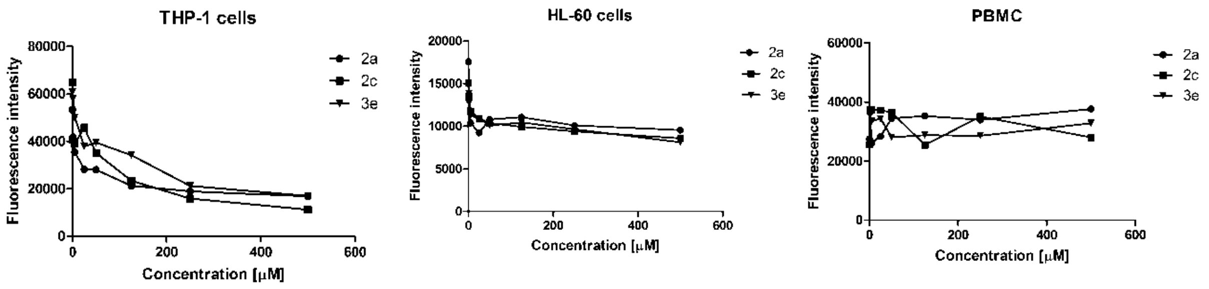

Figure S1. In vitro cell metabolic activity in populations of THP-1, HL-60, and PBMC cell cultures treated with N-(phenothiazinyl)-thioamides **2a, 2c**, and TAPTZ **3e**, monitored by Alamar Blue staining and measurement of fluorescence intensity at 620 nm
